# Peripheral blood flow estimated by laser doppler flowmetry provides additional information about sleep state beyond that provided by pulse rate variability

**DOI:** 10.3389/fphys.2023.1040425

**Published:** 2023-01-26

**Authors:** Zhiwei Fan, Yoko Suzuki, Like Jiang, Satomi Okabe, Shintaro Honda, Junki Endo, Takahiro Watanabe, Takashi Abe

**Affiliations:** ^1^ International Institute for Integrative Sleep Medicine (WPI-IIIS), University of Tsukuba, Tsukuba, Japan; ^2^ The Japan Society for the Promotion of Science (JSPS) Foreign Researcher, Tokyo, Japan; ^3^ Graduate School of Comprehensive Human Science, University of Tsukuba, Tsukuba, Japan; ^4^ KYOCERA Corporation, Kyoto, Japan

**Keywords:** blood flow, pulse rate variability, heart rate variability, sleep stages, respiration, autonomic nervous activity

## Abstract

Pulse rate variability (PRV), derived from Laser Doppler flowmetry (LDF) or photoplethysmography, has recently become widely used for sleep state assessment, although it cannot identify all the sleep stages. Peripheral blood flow (BF), also estimated by LDF, may be modulated by sleep stages; however, few studies have explored its potential for assessing sleep state. Thus, we aimed to investigate whether peripheral BF could provide information about sleep stages, and thus improve sleep state assessment. We performed electrocardiography and simultaneously recorded BF signals by LDF from the right-index finger and ear concha of 45 healthy participants (13 women; mean age, 22.5 ± 3.4 years) during one night of polysomnographic recording. Time- and frequency-domain parameters of peripheral BF, and time-domain, frequency-domain, and non-linear indices of PRV and heart rate variability (HRV) were calculated. Finger-BF parameters in the time and frequency domains provided information about different sleep stages, some of which (such as the difference between N1 and rapid eye movement sleep) were not revealed by finger-PRV. In addition, finger-PRV patterns and HRV patterns were similar for most parameters. Further, both finger- and ear-BF results showed 0.2–0.3 Hz oscillations that varied with sleep stages, with a significant increase in N3, suggesting a modulation of respiration within this frequency band. These results showed that peripheral BF could provide information for different sleep stages, some of which was complementary to the information provided by PRV. Furthermore, the combination of peripheral BF and PRV may be more advantageous than HRV alone in assessing sleep states and related autonomic nervous activity.

## 1 Introduction

Human sleep is a central nervous system (CNS) phenomenon (de Zambotti et al., 2018) and is often analyzed in sleep-wake stages according to the American Academy of Sleep Medicine (AASM) criteria: wakefulness (Wk), three stages of non-rapid-eye-movement (NREM; N1, N2, and N3) sleep, and rapid-eye-movement (REM) sleep (Iber et al., 2007). Different sleep stages correspond to different CNS activity patterns, most commonly measured using an electroencephalogram (EEG). Meanwhile, autonomic nervous system (ANS) activity varies across EEG-defined sleep stages because of the CNS-ANS coupling (de Zambotti et al., 2018). Due to this, sleep state can also be evaluated by assessing ANS activity.

Heart rate and heart rate variability (HRV) derived from an electrocardiogram (ECG) are the most widely used indicators of ANS activity during sleep (Shaffer and Ginsberg, 2017; de Zambotti et al., 2018). Sleep staging using HRV alone has improved its accuracy in previous studies (Imtiaz, 2021). Pulse rate variability (PRV) is another surrogate parameter for HRV, and has even been proposed to be a new biomarker (Yuda et al., 2020). PRV uses techniques such as photoplethysmography (PPG) or laser Doppler flowmetry (LDF) and is derived from signals recorded from peripheral sites, such as fingers and ears, making it even more convenient to measure than ECG-derived HRV (Allen, 2007; Humeau et al., 2009; Tankanag et al., 2020). Sleep assessment using these techniques provides low-cost, automatic, unobtrusive, and home-based alternatives to the gold standard, polysomnography (PSG; including EEG, electrooculogram, and electromyogram), in terms of usability, if not yet of accuracy (Imtiaz, 2021), and has many advantages, especially during the ongoing COVID-19 pandemic, when home-based care has become indispensable (Ueafuea et al., 2021).

However, PRV/HRV do have certain limitations with regard to the assessment of ANS activity and their use for sleep staging. For example, the estimation of cardiac sympathetic modulation by PRV/HRV power in the low-frequency (LF) range (0.04–0.15 Hz) is controversial, although the power in the high-frequency (HF) range (0.15–0.40 Hz) clearly reflects the vagal modulation. The lack of mono-measurement of sympathetic modulation may weaken the accuracy of sleep staging through ANS activity measured by PRV/HRV. In addition, the differentiation ability of HRV differs between specific pairs of sleep stages—for example, it is low between N1 and REM sleep—which further lowers the overall accuracy of sleep staging using HRV (Mitsukura et al., 2020). Meanwhile, PRV cannot identify all the sleep stages (Imtiaz, 2021). Due to these considerations, previous studies on sleep staging utilizing PRV/HRV often included other sources of physiological signals, like body movements, to increase classification accuracy (Imtiaz, 2021); however, this necessitates using other sensing technologies. Conversely, LDF can measure blood flow (BF) besides deriving PRV, but no studies have combined the two for assessing sleep state (Imtiaz, 2021).

LDF-estimated BF may provide additional information about sleep state beyond that provided by PRV/HRV. ANS activity and related processes during sleep may affect peripheral BF and PRV/HRV differently. The two components of the ANS, the parasympathetic and the sympathetic systems, have different roles in the cardiovascular system. While the parasympathetic nervous system contributes to heartbeat activity (Laborde et al., 2017), the sympathetic nervous system (SNS) plays a role in heartbeat activity as well as a dominant role in regulating vascular activity (Task Force of the European Society of Cardiology and the North American Society of Pacing and Electrophysiology, 1996; Lombard and Cowley, 2012). Therefore, peripheral BF may be affected by the same factors like ANS activity that also affect PRV/HRV, through the blood vessel network from the heart, but more by SNS activity and by factors that affect vascular activity alone, such as local (metabolic, myogenic, and paracrine) controls (Sparks, 1999). Thus, some of the variabilities in peripheral BF signals may come from sources that do not affect PRV/HRV in the same way. In addition, peripheral BF signals may vary even more than PRV/HRV due to the modulation by respiration (Khandoker et al., 2011; Dehkordi et al., 2013; Liu et al., 2017). For example, obstructive respiratory events in sleep apnea lead to more changes in the pulse wave amplitude of peripheral BF than in the heart rate derived from an ECG, which shows the effect of respiration on peripheral vascular activity. Further, the SNS affects respiration-modulated BF oscillations in the peripheral vessels through vasomotion (Tankanag et al., 2020). Thus, peripheral BF may have distinct characteristics during sleep and is worth investigating, as examining the dynamics of peripheral BF may reveal its potential for determining and predicting CNS-pattern-defined sleep stages. However, few studies have suggested this possibility while providing complete information on the differences between the five sleep stages (Noll et al., 1994; Shiihara et al., 1999; Kobayashi et al., 2003).

Therefore, the main purpose of this study was to investigate whether and how peripheral BF can differ between the five sleep stages. We hypothesized that peripheral BF would provide information that can be used to distinguish between the sleep stages. We expect that peripheral BF and PRV measured from a single source will have advantages in sleep stage assessment, which may have considerable implications in consumer-grade sleep healthcare at home and in daily situations. As the device used to measure peripheral BF in this study is a commercial device that is still under development, we used PRV results for the validation of BF-based assessments regarding the differences between sleep stages.

## 2 Methods

### 2.1 Participants

All individuals recruited and screened for inclusion in this study self-reported as being physically and psychologically healthy. The included participants satisfied the following criteria: 1) age between 20 and 60 years; 2) able to fill out the Japanese instruction documents, consent forms, and survey forms; 3) able to sleep in the examination rooms in the sleep lab; and 4) not currently being treated for any sleep disorder. Participants were excluded if they were claustrophobic; had uncontrolled diabetes; had a history of myocardial infarction; had unstable angina, serious liver disease, or serious renal disease; were pregnant or may have become pregnant; were lactating; or were judged by the investigator to be inappropriate as participants. On the day of the experiment, participants were also told to not consume alcohol and report any medicine they had taken. The Ethics Committees of the University of Tsukuba (ID: R01-101) approved the research protocol. All participants provided written consent and received payment for participation.

### 2.2 Apparatus and procedure

**FIGURE 1:**
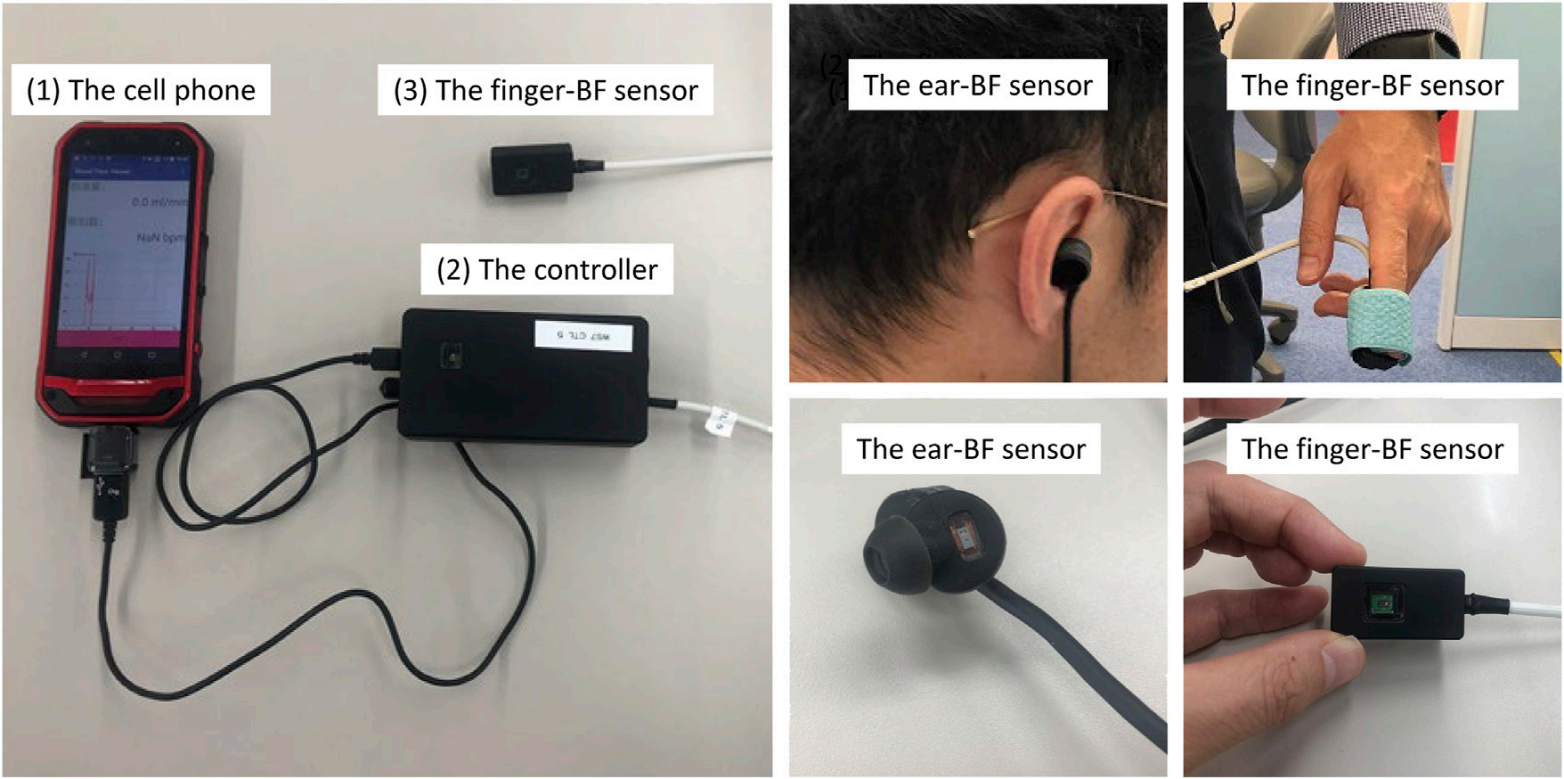
The devices used to measure finger- and ear- BF.

All participants who passed the screening stage underwent an 8-h whole-night sleep in a sound-proof chamber, and their physiological activities were recorded while they slept. PSG and ECG were performed, and BF, respiratory activity, and oxygen saturation (SpO2) were measured. PSG was performed using the PSG-1100 system (Nihon Kohden, Inc., Tokyo, Japan) according to the AASM standards, with the sampling rate set at 250 Hz. For peripheral BF measurement, customized sensors (KYOCERA Corporation, Kyoto, Japan) with a sampling rate of 39.0625 Hz were placed on the right-index finger and the right-ear concha. The sensors had been pre-calibrated to a laser BF meter (RBF-101, PIONEER Corporation, Tokyo, Japan). The originally measured signals are ratios that are then calibrated by this medical BF meter to derive the BF signals (ml/min). The measurement of peripheral BF was based on the LDF method. LDF provides a continuous estimate of skin BF restricted to the skin microcirculation and can be performed in different areas and on different surfaces (Chaseling et al., 2020). The customized sensors used in this study can simultaneously estimate heartbeat activity and peripheral BF (and the derived PRV), thus providing more information than PPG devices. One of the novel aspects and advantages of these sensors is their small size, which makes them convenient for measuring BF on small areas like the concha. Although the sensors’ sampling rate is relatively lower than that of most recent PPG devices (Korkalainen et al., 2020; Wu et al., 2020; Altini and Kinnunen, 2021), they meet the requirement of this study, considering that the spectral analysis was focused on less than 1 Hz. Cellphones were used to store the BF data, which were then exported to a computer for further analysis (Figure 1).

Respiratory activity was measured using a thermistor airflow (AF) sensor, a nasal pressure cannula, and a chest/abdomen band sensor compatible with the PSG-1100. SpO2 was also measured using a sensor compatible with the PSG-1100. Additionally, we validated the accuracy of actigraphy (MotionWatch 8, CamNtech, Fenstanton, United Kingdom) compared to PSG (published elsewhere). Participants were instructed to complete questionnaires about their sleep habits before sleeping. After waking up, they completed a questionnaire about their experience of wearing the ear-BF sensor. Only PSG, ECG, BF, and AF data were used in this study. The data were analyzed using MATLAB (The MathWorks Inc., Natick, MA, USA), R (R Foundation for Statistical Computing, Vienna, Austria), and JASP (0.12.2; The JASP Team, Amsterdam, Netherlands).

### 2.3 Data acquisition and preprocessing

The sleep study was conducted between 8:00 p.m. and 8:00 a.m. The average ambient temperature and humidity for all participants were 22.8°C ± 1.4°C and 61.0 ± 12.6%, respectively. PSG, finger-BF, ear-BF, AF, and other physiological activities were measured simultaneously from the same start time, calibrated across devices. The data points were aligned between these four signals according to the start time and sampling rate. However, considering the availability of the data measured from the finger and ear sites, some participant data were excluded from further analysis. The exclusion criterion was less than 2 h of BF data recorded at night.

PSG data recorded during the 8-h sleep after lights out were analyzed at 30-s epoch by 30-s epoch (in total, 960 epochs for each participant) by a registered polysomnographic technologist (Y. S.) for the sleep stages Wk, N1, N2, N3, and REM according to the AASM criteria (Iber et al., 2007). Finger- and ear-BF data and ECG data were analyzed based on EEG-defined sleep stages. However, considering the epoch length, most HRV indices require epochs longer than 1 min to estimate heart rate (Shaffer and Ginsberg, 2017). We wanted to use epochs that were as short as possible, while at the same time being long enough for all important parameters to be calculated. Therefore, we analyzed data based on 90-s epochs (in total, 320 epochs for each participant); that is, one 90-s epoch contained three consecutive 30-s epochs within the same sleep stage. Three consecutive epochs in the same sleep stage can reflect a relatively stable sleep stage, thus ensuring a stable relationship between the ANS and CNS in the same sleep stage. However, in the case of the three non-consecutive epochs, the 90-s epochs were set to be NaNs (not a number).

Before calculating the PRV/HRV indices, 90-s BF/ECG epochs from each participant were preprocessed using a customized program in MATLAB (see Supplementary Figure S1). Epochs were excluded from further analysis if they met the following criteria: 1, contained extreme raw signal amplitudes (distributed beyond three standard deviations [SDs] of the median); 2, contained extreme peak pulse wave amplitudes (distributed beyond three SDs of the median); 3, contained wrongly detected peaks for more than 10% of the total detected peaks of the pulse waves. These criteria were used to ensure that the 90-s epochs contained no significant artifacts, only pulsate signals with acceptable quality for peak detection and PRV/HRV calculation.

Data were first detrended and filtered using a one-dimensional median filter to remove the spike noise during the recording. Epochs with large amplitudes beyond three SDs of the median value of the highest amplitudes were excluded to remove epochs with significant artifacts. In addition, epochs with peaks of the heartbeat/pulse wave of BF/ECG (filtered using the default band-pass filter of 0.5–2 Hz embedded in the FieldTrip toolbox) (Oostenveld et al., 2011) beyond three SDs of the median value of all the peaks were also excluded to ensure that the quality of the beat signal was optimal. The epochs were then visually inspected to confirm their quality. The usability of the data after the preprocessing is shown in Supplementary Table S1.

To derive the inter-beat intervals (IBIs) from the BF/ECG signals, peak detection was conducted on the waveforms of both ECG and BF signals using the “findpeaks” function in MATLAB. A pre-detection was first conducted to determine the parameter “MinPeakDistance” in “findpeaks”. ECG/BF signals were filtered using the default band-pass filter embedded in the FieldTrip toolbox with a band-pass frequency of 0.5–1.5 Hz. Peak detection was then performed using the default setting of “findpeaks” to determine the peaks and their locations (timings) from the filtered ECG/BF signals. For both BF and ECG signals, “MinPeakDistance” was set to 0.6*mean IBIs (differences in peak timings). BF signals were first filtered using the default high-pass filter embedded in the FieldTrip toolbox with a high-pass frequency of 0.5 Hz for the formal peak detection. After that, the signals were filtered using the “designfilt” function in MATLAB and a twelve order Butterworth low-pass filter with a cutoff frequency of 2.9297 Hz (0.15*sampling rate/2). We then used “findpeaks” to detect the peaks and their locations from the filtered BF signals, with “MinPeakDistance” determined using pre-detection, and “MinPeakHeight,” another parameter, set to zero. ECG signals were decomposed down to level 5 using the default ‘sym4’ wavelet by the “modwt” function in MATLAB and then reconstructed by the “imodwt” function in MATLAB using only the wavelet coefficients at scales 4 and 5 (https://ww2.mathworks.cn/help/wavelet/ug/r-wave-detection-in-the-ecg.html). This was done to enhance the R peaks in the ECG waveform. The reconstructed ECG signals were then subjected to peak detection using “findpeaks” with “MinPeakDistance” determined using pre-detection and “MinPeakHeight” set to 0.15*maximum amplitude of the reconstructed signals. These parameter settings were optimal for peak detection in this study, as confirmed by a visual inspection of the detection results. Epochs with wrong peak detections of more than 10% (average of three wrong detections within one 30-s epoch) were excluded manually (Supplementary Table S2). Finally, ectopic BF IBIs (ECG IBIs were found to be of good quality during manual removal of wrong peak detections), which varied by more than 20% from the previous one were replaced with the mean value of the four neighboring IBIs centered on the ectopic one; this was based on the method typically adopted in clinical practice and human and animal research for HRV analysis (Altini et al., 2016; Betti et al., 2018; Karey et al., 2019). However, the first two were replaced with the mean value of the four subsequent IBIs, whereas the last two were replaced with the mean value of the four previous IBIs, a method similar to that used in a previous study (Karey et al., 2019).

We then calculated time-domain, frequency-domain, and non-linear PRV/HRV metrics related to ANS activity (Shaffer and Ginsberg, 2017). We selected several commonly used parameters in all three domains (Supplementary Table S3). In the time domain, the SD of all the normal-to-normal (NN) intervals (SDNN; i.e., typical IBIs resulting from sinus node depolarization, which are free from artifacts), the root mean square of successive differences between adjacent NN intervals (RMSSD), and percentage of pairs of adjacent NN intervals differing by more than 50 ms (pNN50) are indices often chosen to reflect ANS activity (Task Force of the European Society of Cardiology and the North American Society of Pacing and Electrophysiology, 1996; Shaffer and Ginsberg, 2017). The latter two indices measure cardiac vagal modulation (Shaffer and Ginsberg, 2017; de Zambotti et al., 2018). In the frequency domain, generally within 0.4 Hz, the HRV spectral power in the very-LF range (<0.04 Hz) is less likely to be interpreted. The HRV spectral power in the LF range (0.04 Hz–0.15 Hz) is often reported to reflect sympathetic activity; however, this conclusion is controversial (de Zambotti et al., 2018). In contrast, the HF range (0.15–0.4 Hz) reflects vagal (parasympathetic) activity (Task Force of the European Society of Cardiology and the North American Society of Pacing and Electrophysiology, 1996; Shaffer and Ginsberg, 2017; de Zambotti et al., 2018). LF and HF are usually normalized by dividing them by the total power (LF + HF). The normalized LF and HF (LFn and HFn) and the LF/HF ratio also reflect ANS activity (Iber et al., 2007; de Zambotti et al., 2018). There are also non-linear measures for assessing ANS activity using HRV and without assuming linearity and stationarity of the IBI time series. Such measures include detrended fluctuation analysis (DFA), which is widely used for detecting short- and long-range correlations in non-stationary time series (Peng et al., 1994; Peng et al., 1995; Shaffer and Ginsberg, 2017), and entropy measures, such as approximate entropy (ApEn) (Pincus, 1991; Shaffer and Ginsberg, 2017; Udhayakumar et al., 2017), which measures the predictability of fluctuations in the time series. These measures may assess non-linear HRV patterns related to more complex functioning, such as sleep. All indices, except for DFA to measure short-range fluctuations (DFA1), DFA to measure short-range fluctuations (DFA2), and ApEn, were calculated using a customized MATLAB script according to their definition. For DFA1, DFA2, and ApEn with more complex calculations, the scripts from the references (Lee, 2021; Magris, 2021) were used.

The power spectra of BF, PRV, and HRV were analyzed using the “plomb” function in MATLAB. Since the HRV and PRV time-series data points were sampled at different times, the frequency coordinates differed among epochs when calculating the power spectra. This made it impossible to average the power spectra across epochs in the same sleep stage or compare the power spectra across sleep stages. Thus, interpolation was conducted after using “plomb” to unify the frequency coordinates. However, interpolation was not conducted on the BF power spectra. The power of a particular band within 0.2–0.3 Hz was extracted, normalized by dividing by the HF, and compared. This frequency band is within the HF band (0.15–0.4 Hz), in which sleep stages modulate HRV power (Naji et al., 2019; Whitehurst et al., 2020). There are three reasons for focusing on this narrower band. First, we noted (without a hypothesis beforehand) that all the group results of the BF, PRV, and HRV spectra showed this peak frequency band. Second, previous studies have shown that stimulation around 0.25 Hz promotes sleep (Bayer et al., 2011; Perrault et al., 2019; van Sluijs et al., 2020). Third, the effects of respiration around 0.2–0.3 Hz on cardiovascular activity have been investigated in previous studies (Krasnikov et al., 2013; Tankanag et al., 2020).

To provide information on the dynamics of BF, typical time-domain parameters, including the mean, SD, and the coefficient of variance (CV) of BF volume (ml/min) during 90-s epochs in different sleep stages, were calculated using the raw, unfiltered BF data, but with epochs corresponding to PRV data after preprocessing. Further, frequency-domain parameters corresponding to PRV, including the LFn, HFn, LF/HF, and normalized power of the 0.2–0.3 Hz band, were also calculated.

For each index calculated for all participants, values larger than three SDs of the group means were set as missing values. Participants with missing values in either of the five stages were excluded from the group analysis. Thus, the group analysis for each dependent variable may have had a different set of participants.

### 2.4 Statistical analysis

We investigated the changes in each BF, AF, HRV, and PRV parameter across the sleep stages separately for each recording site (heart, finger, or ear) using the Bayesian analysis approach. The Jarque-Bera test (Jarque and Bera, 1987) with a significance level of 0.01 was used to test the normality of the distribution of each index across individuals. For indices that met the normality criteria, we performed parametric analysis. The Bayesian Wilcoxon signed-rank test was used as a non-parametric version of Bayesian pairwise comparison for indices that did not meet the normality criteria. The Bayes factors were derived and interpreted using a classification scheme (Kass and Raftery, 1995; Lee and Wagenmakers, 2013; Quintana and Donald, 2018). The advantage of using the Bayes factor is that it shows the amount of evidence for the null hypothesis (H0) or the alternative hypothesis (H1) or insufficient evidence for either hypothesis (Dienes, 2016). Therefore, the Bayes factor can provide the amount of evidence for H1 against H0 (or H0 against H1). For example, the Bayes factor $B_{10}$ shows the level of the possibility of H1 against H0, and its value is classified into different categories of evidence (Supplementary Table S4, adapted from Lee and Wagenmakers, 2013; Quintana and Donald, 2018). We also used the frequentist test to obtain complementary information (Supplementary Table S5, S6).

## 3 Results

Data were obtained from 45 participants (13 women; mean age, 22.5 ± 3.4 years). Specifically, the finger-BF data of 38 participants (10 women; mean age, 22.6 ± 3.7 years), ear-BF data of 42 participants (13 women; mean age, 22.6 ± 3.5 years), and the PSG, ECG, and AF data of 45 participants remained after applying the exclusion criteria. In addition, for each index investigated as follows, values larger than three SDs of the group means were set as missing values, and participants with missing values in either of the five stages were excluded from the group analysis. Thus, the group analysis for each dependent variable may have a different set of participants. For example, for mean heat-IBI, data of only 40 participants (12 women) remained for group analysis.

### 3.1 Comparison of the changes in parameters of finger- and ear-BF signals across different sleep stages

#### 3.1.1 Time-domain indices

**FIGURE 2:**
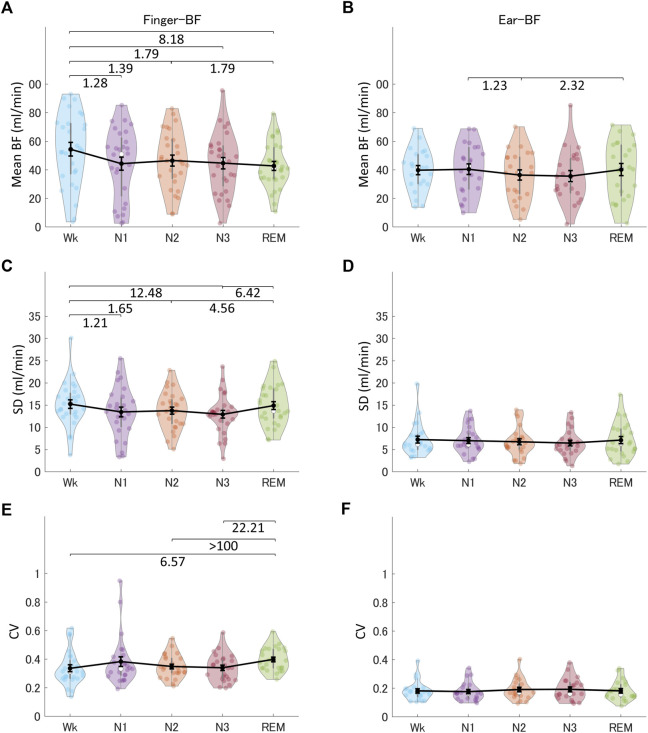
The indices of finger-BF and ear-BF in the time domain, including the mean **(A, B)**, SD **(C, D)**, and CV **(E, F)** of BF, across the different sleep stages. The violin plot with dots shows the distribution of the individual data points. The line chart with error bars shows the group mean and the ±1 standard error of the mean. The numerical values are the Bayes factors. The values show anecdotal (1–3), moderate (3–10), strong (10–30), or extreme evidence (>100) against the H0 of no difference between pairs of sleep stages (for other information, see Supplementary Table S6). Bayes factors <1 are not listed. BF, blood flow; SD, standard deviation; CV, coefficient of variance.

We investigated the time-domain and frequency-domain (in the following paragraphs) parameters of finger-BF and ear-BF, expecting that BF signals would provide information for the differentiation of sleep stages. Figure 2 shows the indices of finger-BF and ear-BF in the time domain, including the mean amplitude, SD, and CV of BF. The results revealed that sleep stages modulated finger-BF. Particularly, finger-BF showed a robust difference in mean amplitude and CV between Wk and REM sleep. The mean finger-BF in Wk was larger than that in REM sleep. In contrast, CV, reflecting the relative fluctuation, was larger in REM sleep than in Wk. However, except for some weak evidence, ear-BF was not significantly modulated by the sleep stages (see also Supplementary Table S6).

#### 3.1.2 Frequency-domain indices

**FIGURE 3:**
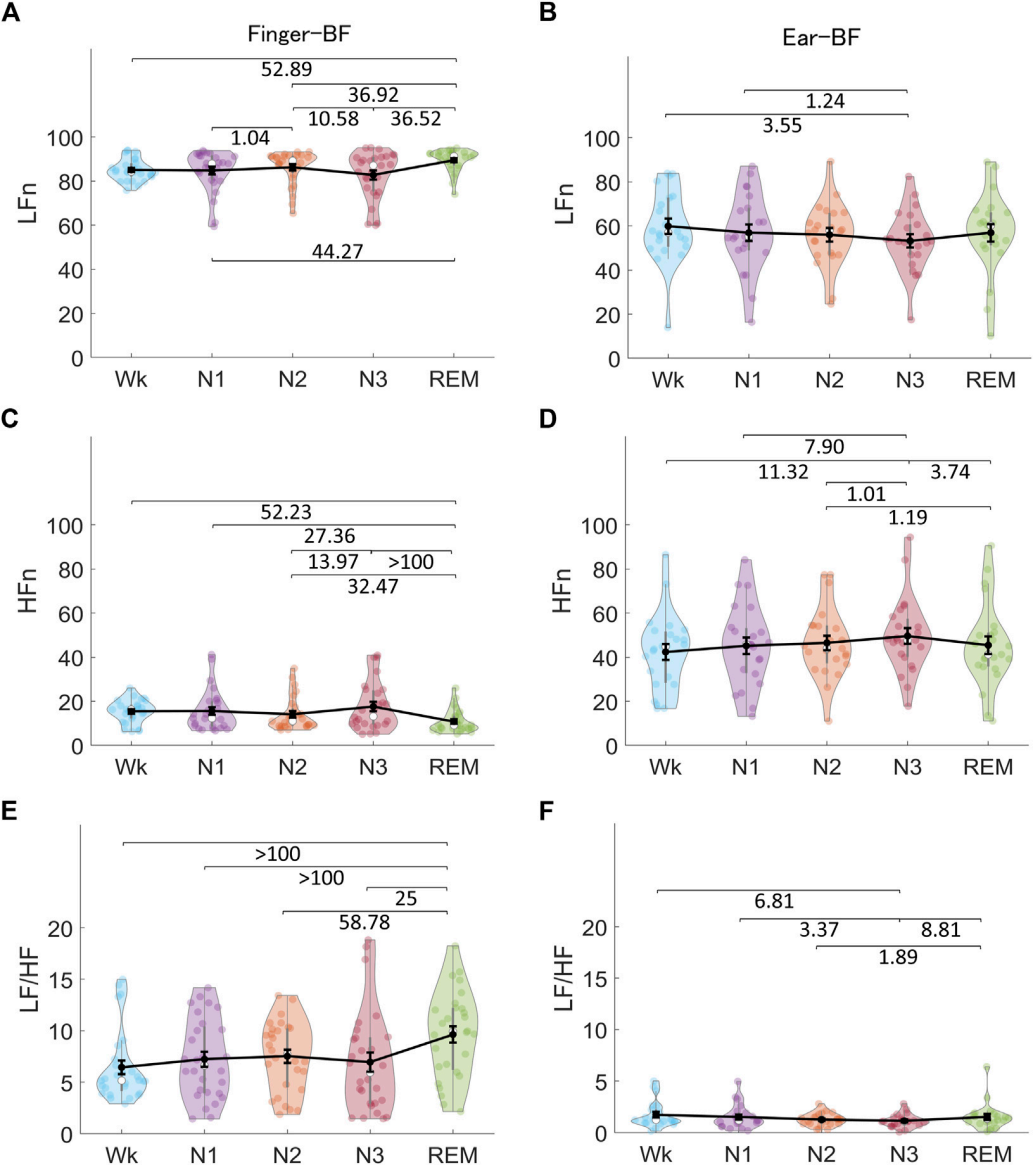
The indices of finger-BF and ear-BF in the frequency domain, including the LFn **(A, B)**, HFn **(C, D)**, and LF/HF **(E, F)** of BF, across the different sleep stages. The violin plot with dots shows the distribution of the individual data points. The line chart with error bars shows the group mean and the ±1 standard error of the mean. The numerical values are the Bayes factors. The values show anecdotal (1–3), moderate (3–10), strong (10–30), very strong (30–100), or extreme (>100) evidence against the H0 of no difference between the pairs of sleep stages (for other information, see Supplementary Table S6). Bayes factors <1 are not listed. LFn, normalized low-frequency power; HFn, normalized high-frequency power; LF, low-frequency power; HF, high-frequency power; BF, blood flow; AF, airflow.

Figure 3 shows the normalized low-frequency power (LFn), normalized high-frequency power (HFn), and low-frequency power/high-frequency power (LF/HF) of raw finger-BF and ear-BF. The results revealed that sleep stages modulated both finger- and ear- BF. Similarly, finger-BF showed robust differences in LFn and HFn between Wk and REM sleep, N1 and REM sleep, and N3 and REM sleep. LFn, HFn, and LF/HF of finger-BF prominently highlighted REM sleep over all other stages. Ear-BF was also modulated by the sleep stages, with N3 being highlighted instead, although the evidence for this was weak.

#### 3.1.3 Comparison of the changes in 0.2–0.3 Hz oscillations of BF spectra across different sleep stages

**FIGURE 4:**
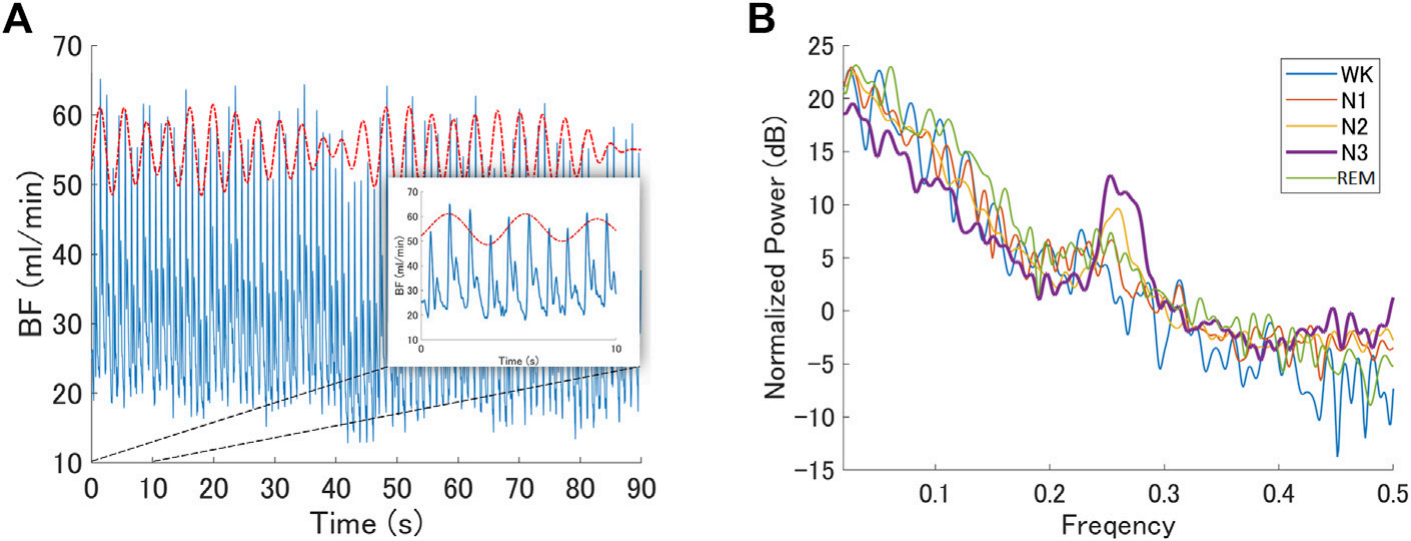
BF modulated by oscillations within the 0.2–0.3 Hz band **(A)** A representative epoch of the raw BF signal modulated by 0.2–0.3 Hz oscillations during N3 recorded from a representative participant. The red dotted curve represents the oscillating signal filtered from the raw BF signal with a band-pass frequency of 0.2–0.3 Hz; however, it has been shifted upwards to make it easier to read. The blue curve is the raw BF signal **(B)** The normalized power spectrum of a representative participant at different sleep stages. It shows a peak in the 0.2–0.3 Hz frequency band during N3. BF, blood flow.

**FIGURE 5:**
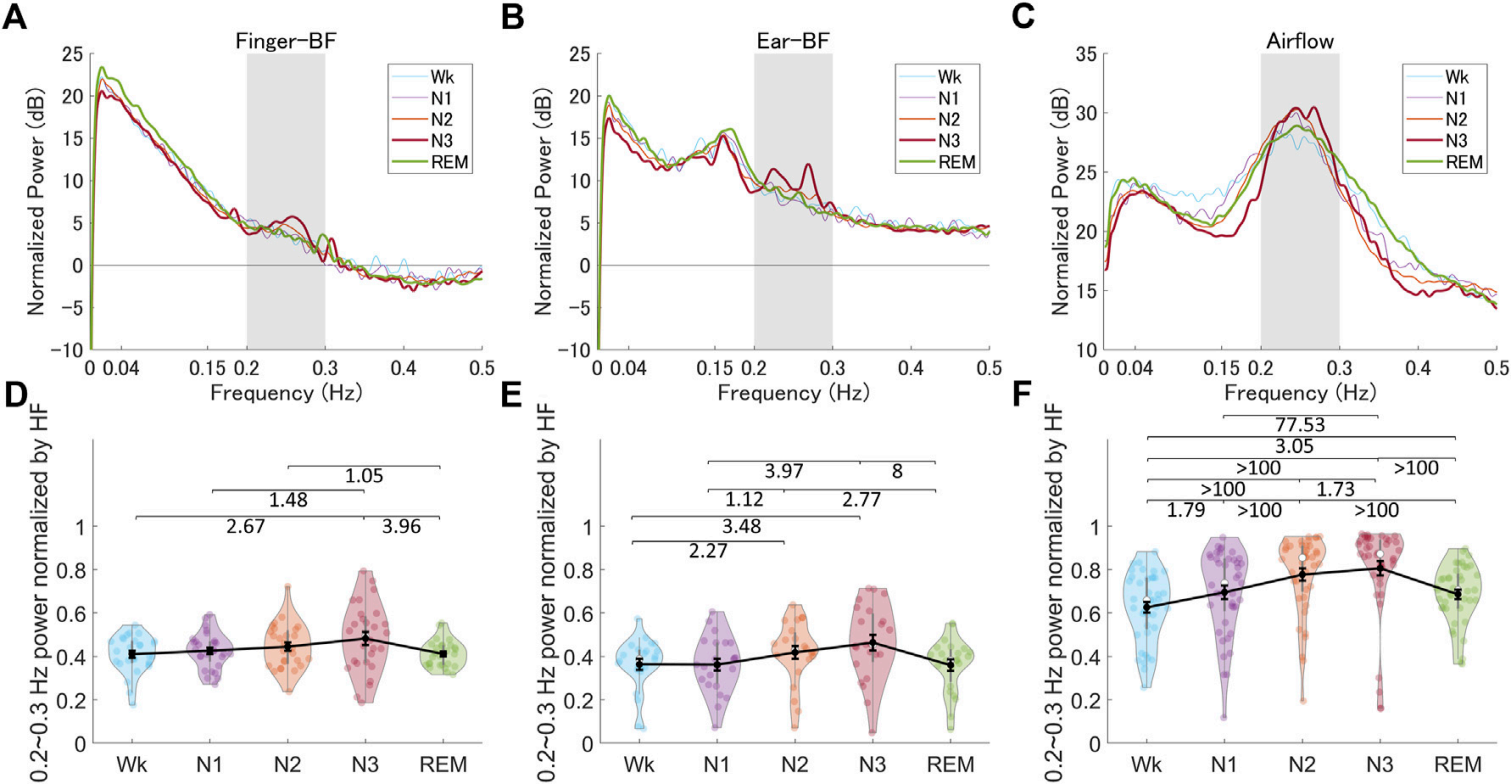
The normalized power spectra and the normalized power in the 0.2–0.3 Hz band of finger-BF, ear-BF, and AF, across the different sleep stages **(A–C)** The normalized power spectra for finger-BF **(A)**, ear-BF **(B)**, and AF **(C) (D–F)** The normalized power of the 0.2–0.3 Hz band for finger-BF **(D)**, ear-BF **(E)**, and AF **(F)**. There was a linear trend of power increase in the 0.2–0.3 Hz band with the deepening of sleep from N1 to N3 for both finger- and ear- BF. The violin plot with dots shows the distribution of the individual data points. The line chart with error bars shows the group mean and the ±1 standard error of the mean. The numerical values are the Bayes factors. The values show anecdotal (1–3), moderate (3–10), very strong (30–100), or extreme (>100) evidence against the H0 of no difference between pairs of sleep stages (for other information, see Supplementary Table S6). Bayes factors <1 are not listed. BF, blood flow; HF, high-frequency power.

We examined a particular BF band within 0.2–0.3 Hz oscillations based on the reasons described in the Methods section. A representative BF signal modulated in a 0.2–0.3 Hz oscillation during N3 is shown in Figure 4A. We noted a peak power within the HF band (0.15–0.4 Hz), specifically around 0.2–0.3 Hz, as shown in Figure 4B; Figures 5A,B show the grand averages of the power spectra of raw finger- and ear-BF, which also had a peak around 0.2–0.3 Hz. Furthermore, the 0.2–0.3 Hz band power was modulated by sleep stages; Figures 5D,E show the power in the 0.2–0.3 Hz band normalized by HF to highlight the peak power for each recording site (referred to as BF_Pow_02_03; see the dark area in Figures 5A,B) across sleep stages. The results showed that for both finger- and ear-BF, BF_Pow_02_03 was higher in N3 sleep than in REM sleep and Wk. In NREM sleep, BF_Pow_02_03 was higher in N3 than in N1; however, the evidence for finger-BF was weak. We then extracted the data for N1–N3 and conducted a trend analysis, one of the sub-analyses of the analysis of variance in JASP. The results showed a linear trend in finger-BF (*p* = 0.001) and ear-BF (*p* = 0.001). Finger-BF was consistent with ear-BF with respect to BF_Pow_02_03 across all three NREM sleep stages; both had the highest value in N3. BF_Pow_02_03 (in 0.2–0.3 Hz) showed its advantage over HFn (in 0.15–0.4 Hz) for highlighting N3, suggesting the necessity of focusing on this narrower 0.2–0.3 Hz band.

### 3.2 0.2–0.3 Hz oscillations of AF spectra across different sleep stages and their correlation with BF

We observed special 0.2–0.3 Hz oscillations in BF within the 0.15–0.4 Hz respiratory frequency band. This band of oscillations may be modulated by respiration activity. Therefore, we analyzed the AF data and investigated the correlation between the BF and AF activities (Supplementary information) to gain more insight into the mechanism underlying the 0.2–0.3 Hz oscillations in BF. Figures 5C,F show the power spectra of AF and the power in the 0.2–0.3 Hz band (referred to as AF_Pow_02_03; see the dark area in Figure 5C), which was normalized by the HF (see Supplementary Figure S2 and Supplementary Table S6 for more information on the LFn, HFn, and LF/HF of AF). The results showed that AF_Pow_02_03 also seemed to be a good indicator for distinguishing between sleep stages. Further, correlations between the 0.2–0.3 Hz power as well as the peak frequency for BF and for AF were significant in several sleep stages, especially in N2 and N3, suggesting a close relationship between peripheral BF and respiration that may be modulated by sleep stage (see Supplementary Figure S3).

### 3.3 Comparison of changes in finger- and ear-PRV and HRV indices across different sleep stages

**FIGURE 6:**
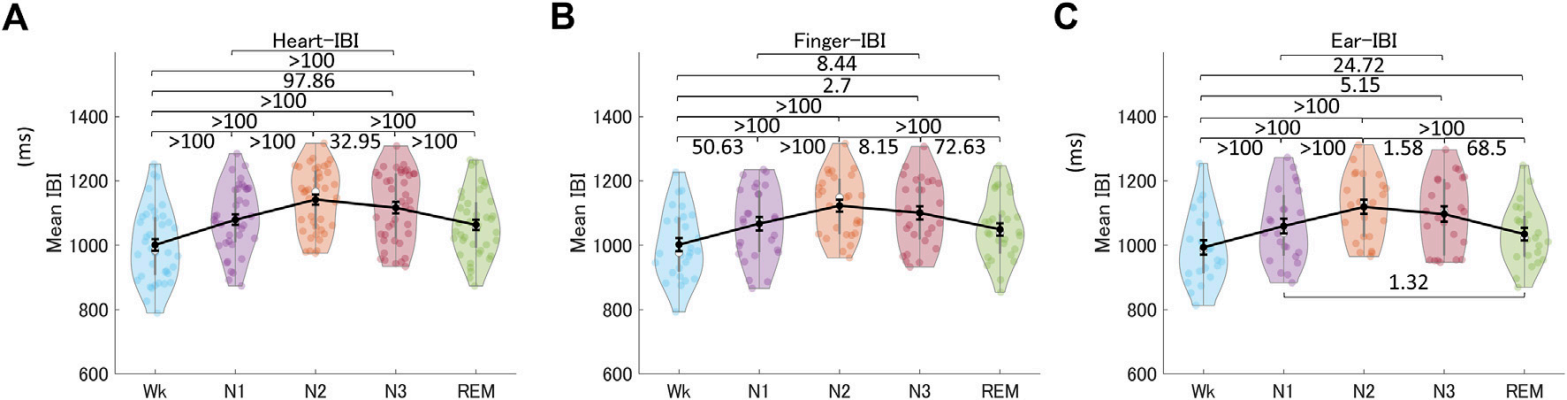
Mean IBIs across the different sleep stages for the heart **(A)**, the finger **(B)**, and the ear **(C)**. The violin plot with dots shows the distribution of the individual data points. The line chart with error bars shows the group mean and the ±1 standard error of the mean. The numerical values are the Bayes factors. The values show anecdotal (1–3), moderate (3–10), strong (10–30), very strong (30–100), or extreme (>100) evidence against the H0 of no difference between pairs of sleep stages (for other information, see Supplementary Table S6). Bayes factors <1 are not listed. IBI, inter-beat interval.

Next, we compared PRV indices derived from peripheral BF across different sleep stages to assess whether they showed similar changes as HRV. Figure 6 shows the mean heart-, finger-, and ear-IBIs across the sleep stages. These results showed that the mean IBI differed across sleep stages, providing information for differentiating all pairs of different sleep stages, except N1 *versus* REM, for all three recording sites (see also Supplementary Table S6). The mean IBI was higher (i.e., heart rate was lower) in NREM sleep (specifically, N2 and N3) than in REM sleep, and in both NREM and REM sleep than in Wk. This revealed similar patterns across the sleep stages for the mean heart-IBI, mean finger-IBI, and mean ear-IBI.

#### 3.3.1 Time-domain indices

**FIGURE 7:**
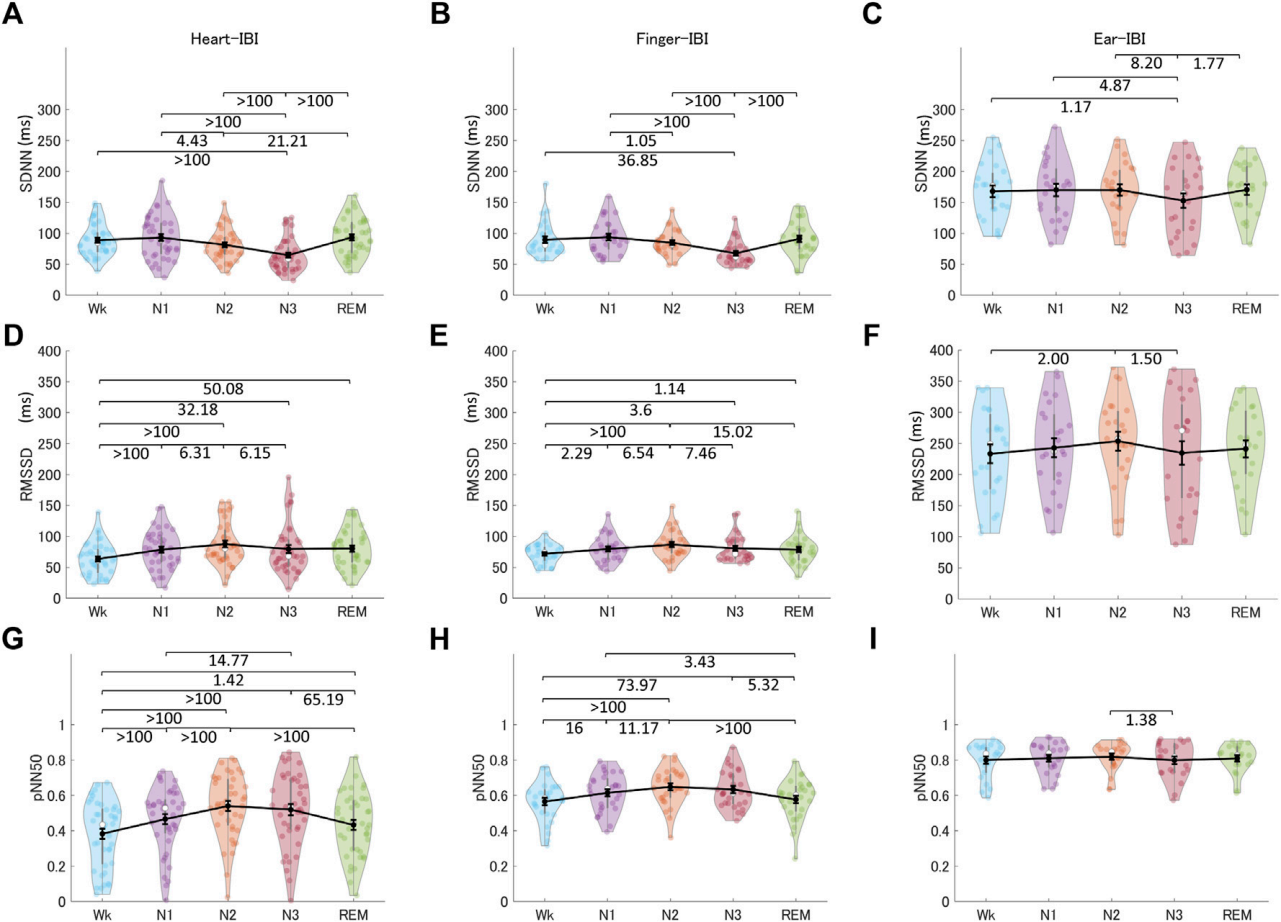
The indices of HRV **(A, D, G)**, finger-PRV **(B, E, H)**, and ear-PRV **(C, F, I)** in the time domain, including SDNN **(A–C)**, RMSSD **(D–F)**, and pNN50 **(G–I)**, across the different sleep stages. The violin plot with dots shows the distribution of the individual data points. The line chart with error bars shows the group mean and the ±1 standard error of the mean. The numerical values are the Bayes factors. The values show anecdotal (1–3), moderate (3–10), strong (10–30), very strong (30–100), or extreme (>100) evidence against the H0 of no difference between pairs of sleep stages (for other information, see Supplementary Table S6). Bayes factors <1 are not listed. HRV, heart rate variability; PRV, pulse rate variability; SDNN, standard deviation of all the normal-to-normal (NN) intervals; RMSSD, root mean square of successive differences between adjacent NN intervals; pNN50, percentage of pairs of adjacent NN intervals differing by more than 50 ms; IBI, inter-beat interval.

Figure 7 shows the HRV, finger-PRV, and ear-PRV indices in the time domain, including SDNN, RMSSD, and pNN50. The PRV/HRV indices in the time domain showed that the finger indices patterns were very similar to the heart indices patterns. Specifically, the SDNN for HRV and finger-PRV were lower in N3 sleep than in REM sleep and Wk. For NREM sleep, SDNN was lower in N3 than in N2 and N1, and lower in N2 than in N1 (weak evidence for finger-PRV). Ear-SDNN showed similar results but with weaker evidence. The RMSSD for HRV and finger-PRV were higher in NREM sleep (N2 and N3) than in Wk. For NREM sleep, moderate evidence observed from Bayes factors showed that heart- and finger-RMSSD were higher in N2 than in N1 and N3. Ear-RMSSD also showed anecdotal evidence to be highest in N2. Similarly, the pNN50 for HRV and finger-PRV were higher during NREM sleep (N2 and N3) than during Wk and REM sleep. For NREM sleep, heart- and finger- RMSSD were higher in N2 than in N1. Overall, the sensitivity of ear-PRV in time-domain indices may be lower than that of HRV or finger-PRV, as reflected by the smaller Bayes factors between the different sleep stages.

#### 3.3.2 Frequency-domain indices

**FIGURE 8:**
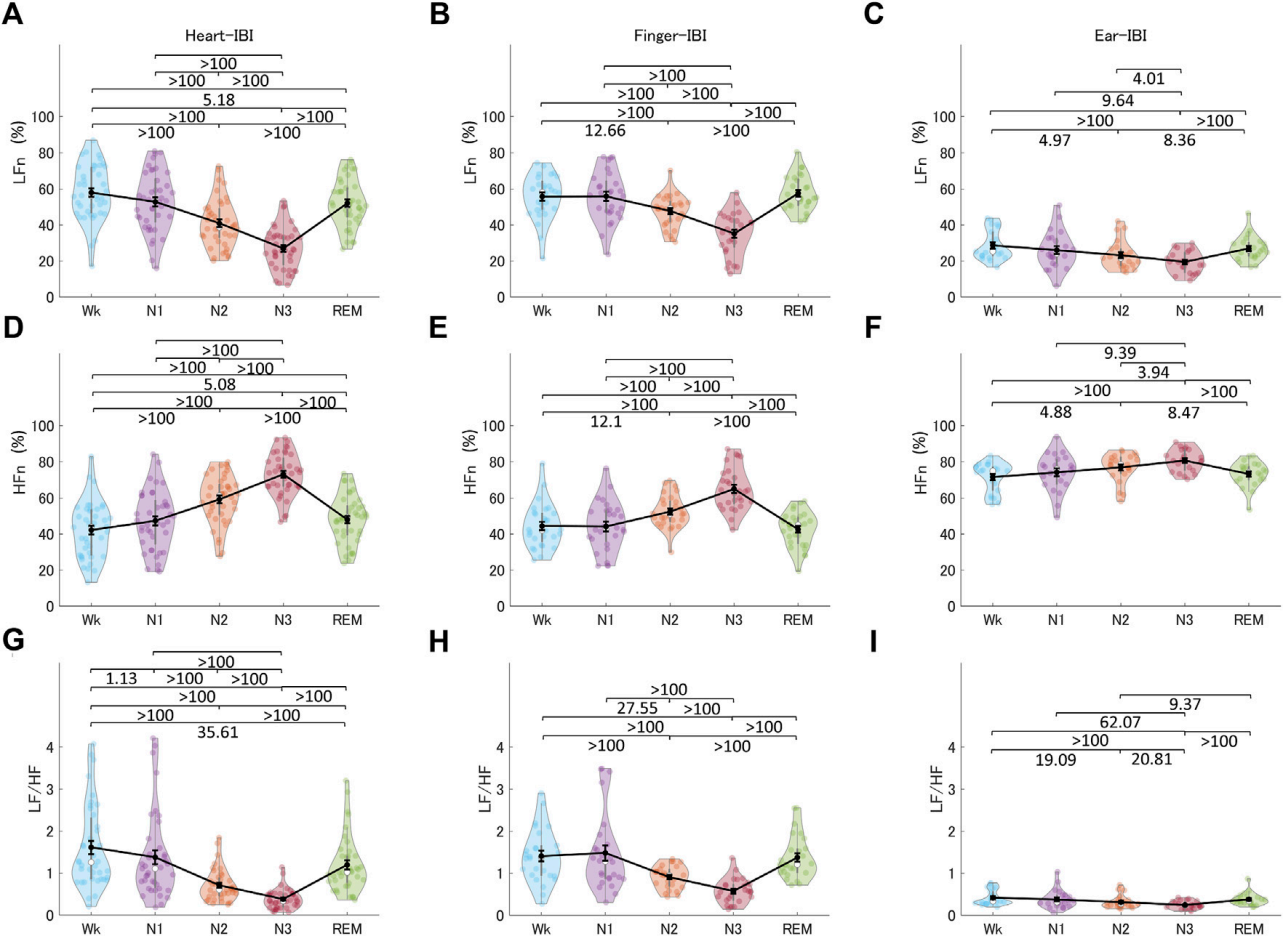
The indices of HRV **(A, D, G)**, finger-PRV **(B, E, H)**, and ear-PRV **(C, F, I)** in the frequency domain, including LFn **(A–C)**, HFn **(D–F)**, and LF/HF **(G–I)**, across the different sleep stages. The violin plot with dots shows the distribution of the individual data points. The line chart with error bars shows the group mean and the ±1 standard error of the mean. The numerical values are the Bayes factors. The values show anecdotal (1–3), moderate (3–10), strong (10–30), very strong (30–100), or extreme (>100) evidence against the H0 of no difference between pairs of sleep stages (for other information, see Supplementary Table S6). Bayes factors <1 are not listed. HRV, heart rate variability; PRV, pulse rate variability; LF, low-frequency power; HF, high-frequency power; LFn, normalized low-frequency power; HFn, normalized high-frequency power; IBI, inter-beat interval.

Figure 8 shows the indices of HRV, finger-PRV, and ear-PRV in the frequency domain, including LFn, HFn, and LF/HF. As with the results of the PRV/HRV indices in the time domain, the finger indices in the frequency domain exhibited very similar patterns to those of the heart indices. The ear indices showed the smaller Bayes factors between sleep stages. Specifically, LFn for HRV, finger-PRV, and ear-PRV were lower in NREM sleep (N2 and N3) than during REM sleep and Wk. For NREM sleep, LFn was lower in N3 than in N2 and N1, and lower in N2 than in N1 (except for ear-LFn). The HFn results were opposite to the LFn results, whereas LF/HF results were similar to the LFn results.

#### 3.3.3 Non-linear measurements

**FIGURE 9:**
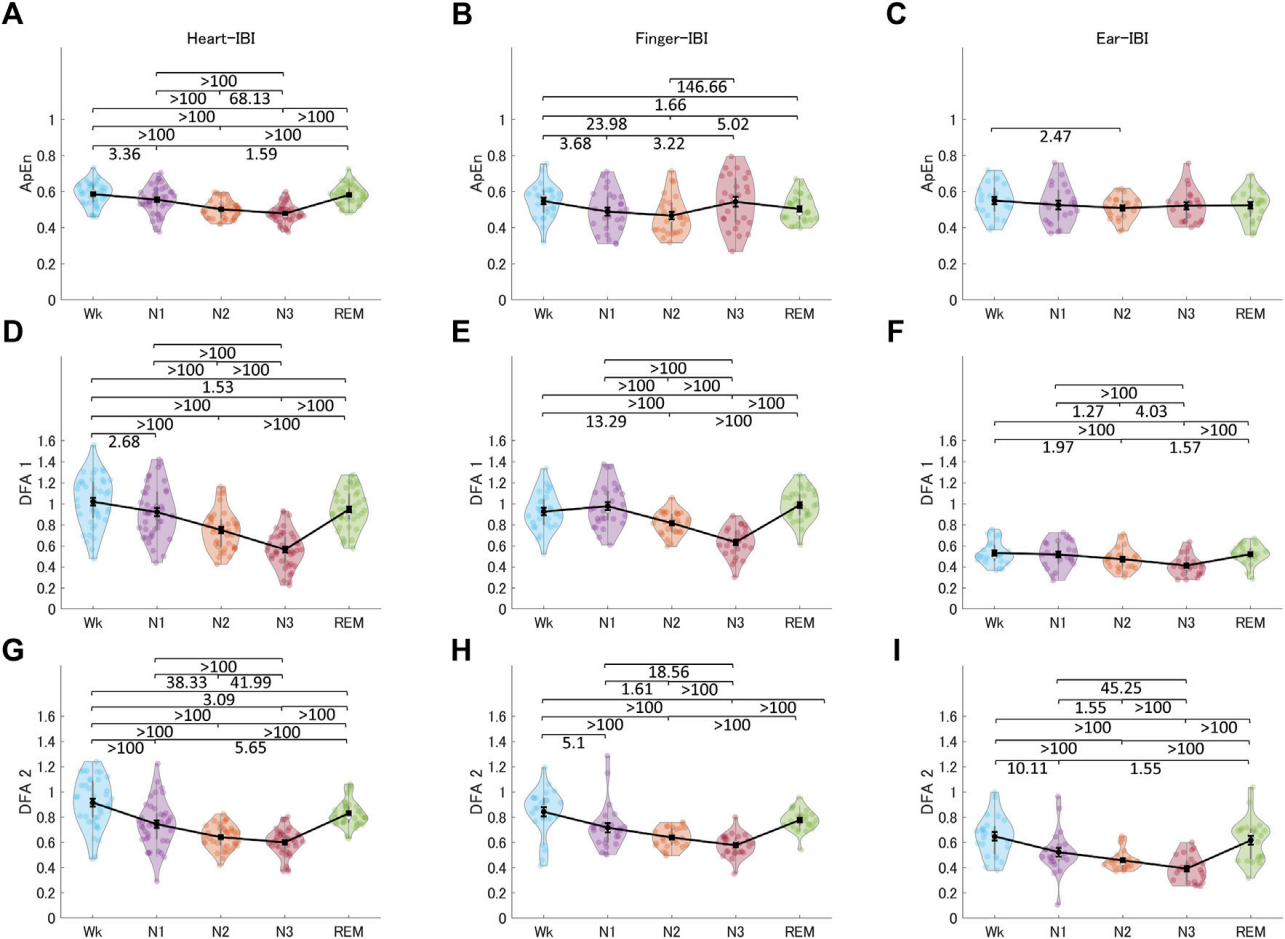
The indices of HRV **(A, D, G)**, finger-PRV **(B, E, H)**, and ear-PRV **(C, F, I)** in non-linear measurements, including ApEn **(A–C)**, DFA1 **(D–F)**, and DFA2 **(G–I)**, across the different sleep stages. The violin plot with dots shows the distribution of the individual data points. The line chart with error bars shows the group mean and the ±1 standard error of the mean. The numerical values are the Bayes factors. The values show anecdotal (1–3), moderate (3–10), strong (10–30), very strong (30–100), or extreme (>100) evidence against the H0 of no difference between pairs of sleep stages (for other information, see Supplementary Table S6). Bayes factors <1 are not listed. HRV, heart rate variability; PRV, pulse rate variability; ApEn, approximate entropy; DFA, detrended fluctuation analysis; IBI, inter-beat interval.

Figure 9 shows the non-linear indices of HRV, finger-PRV, and ear-PRV, including ApEn, DFA1, and DFA2. The non-linear finger and ear indices (DFA1 and DFA2, other than ApEn) showed very similar patterns to those of the heart indices. Specifically, ApEn for HRV and finger-PRV were lower in NREM sleep (N1 and N2) than in Wk and lower in N2 than in REM sleep. However, in NREM sleep, evidence showed that finger-ApEn was higher in N3 than in N2 and N1, which is contrary to the result for heart-ApEn. DFA1 and DFA2 showed similar results. For all three recording sites, DFA1 and DFA2 were lower in NREM sleep (N2 and N3) than in REM sleep and Wk; however, the ear-PRV indices showed weaker evidence. In NREM sleep, they were lower in N3 than in N2 and N1, and in N2 than in N1. In contrast, DFA1 showed higher values in N1 than in Wk for finger-PRV with moderate evidence.

## 4 Discussion

This study investigated peripheral BF and PRV across different sleep stages and compared PRV with HRV during sleep. The time- and frequency-domain parameters of peripheral BF could also provide information regarding the different sleep stages, especially REM sleep. We observed significant peaking of oscillations around 0.2–0.3 Hz, especially during N3, for both the peripheral BF signal and the derived IBI signal (see Supplementary Figure S4). For finger- and ear-BF, finger- and ear-PRV, and HRV, there was an increase in oscillations within the 0.2–0.3 Hz band with the deepening of NREM sleep (N1 to N3). This increase was highest in N3. Comparisons of the time-domain, frequency-domain, and non-linear PRV/HRV indices across the sleep stages revealed that the patterns of finger-PRV indices were consistent with those of most HRV indices, especially for the time-frequency-domain indices. This confirmed that BF and PRV were being measured correctly by the customized sensors used in this study. However, differences existed between finger-PRV and HRV indices, especially in non-linear metrics such as ApEn. Ear-PRV could also provide information for differentiating sleep stages, comparable to the information provided by some of the HRV indices. In some respects, peripheral BF + PRV may provide more information than HRV alone. Therefore, further investigation is required on the potential of peripheral BF + PRV for sleep state assessment.

### 4.1 Peripheral BF dynamics across sleep stages

Normal human sleep is associated with hemodynamic changes, primarily mediated by changes in the ANS. Specifically, during NREM sleep, sympathetic activity decreases, and parasympathetic (vagal) activity increases with the development of slow-wave sleep (N3); however, both these changes are reversed during REM sleep (Trinder et al., 2001; Narkiewicz et al., 2016). These distinct changes during different sleep stages are reflected in heart rate and PRV/HRV calculated from the IBI signal. In the present study, the mean IBI, an index of changes in the balance of the two ANS components, increased (i.e., heart rate decreased) during NREM sleep, which deepens from N1 to N3. However, it decreased during REM sleep (more specifically, phasic REM sleep) but remained higher than the mean IBI in Wk (Somers et al., 1993; Trinder et al., 2001; Hanak and Somers, 2011). The mean finger- and ear-IBIs showed differences between nearly all pairs of different sleep stages except for N1 and REM sleep.

Sleep stages also modulate PRV indices with different ANS activity patterns. Frequency-domain indices, including LFn, HFn, and LF/HF, showed nearly the same patterns across sleep stages as the mean IBI. They also showed differences between most pairs of different sleep stages, except between Wk, N1, and REM sleep. Although LF reflects a mix of sympathetic and parasympathetic power and HF reflects the vagal tone, the calculation of LFn, HFn, and LF/HF makes them equivalent in terms of information on the patterns of ANS activity across sleep stages (Laborde et al., 2017; de Zambotti et al., 2018). Time-domain indices, including pNN50, are correlated with RMSSD and HF; therefore, the two indices in the time domain (for finger-PRV) showed similar patterns. In contrast, LF contributes significantly to SDNN; thus, the SDNN pattern for finger-PRV was similar to that of LFn (de Zambotti et al., 2018). The non-linear indices of both finger- and ear-PRV, specifically DFA1 and DFA2, measure the fluctuations of IBIs. Thus, they provide as much information for differentiating between sleep stages as the frequency-domain indices that measure the oscillations of IBIs. The patterns of HRV/finger-PRV indices (including SDNN, LFn, HFn, and LF/HF) were consistent with those reported in a recent study (Liu et al., 2017). However, that study did not provide significant test results for the comparisons between the different sleep stages.

Besides PRV, the ANS may modulate peripheral BF differently during different sleep stages. For example, the peripheral arterial tone (PAT) signal from BF, an index of sympathetic vasoconstrictor mechanisms (Pépin et al., 2009; Lanfranchi and Somers, 2010), decreases from Wk to NREM sleep and reaches its nadir during REM sleep (Lavie et al., 2000). The decreasing pattern of the PAT signal suggests a similar decreasing pattern for BF across sleep stages because they share similarities in the assessment of peripheral pulse waves (Grote and Zou, 2017). The mean amplitude of peripheral BF in the present study confirmed that skin BF at the finger gradually decreases from Wk, to NREM sleep, and to REM sleep (see Figure 2A). In addition, because REM sleep is associated with a largely variable sympathetic tone (Lanfranchi and Somers, 2010), peripheral BF during this stage should be variable as well. This was also confirmed by the CV and frequency-domain results of finger-BF in the present study. Furthermore, previous studies have shown that the modulation of BF by sleep stages (e.g., REM sleep with higher sympathetic activity) may be different between the peripheral and cerebral regions. While peripheral BF may decrease in REM sleep compared with NREM sleep (Lavie et al., 2000), cerebral BF shows a marked increase instead (Hanak and Somers, 2011; Klingelhófer, 2012). Therefore, peripheral BF has its own characteristics during sleep, and these characteristics may be different across sleep stages. Indeed, compared with PRV in the present study, peripheral BF activity was highlighted in REM sleep. In particular, peripheral BF provided distinct information for the differences between REM sleep and other stages, such as Wk and N1, which all PRV indices and most HRV indices could not provide. Peripheral BF + PRV may be able to differentiate all the sleep stages and provides good indices for measuring ANS activity, including parasympathetic and sympathetic activity. In some respects, they may compare favorably with HRV because HRV indices (e.g., LFn) do not have mono-measures for sympathetic tone. However, peripheral BF indices in the time-frequency domain may be candidates for assessing SNS activation.

### 4.2 Similarities and differences between PRV and HRV regarding ANS activity during sleep

With every heartbeat, blood is transferred from the heart through the blood vessel network to peripheral areas, such as fingers. Because of this close relationship, previous studies have attempted to reconstruct ECG signals from the pulse wave signals of blood in tissues (Zhu et al., 2019). Although many external factors can affect the consistency between PRV and HRV, the physiological natures of peripheral BF and ECG signals leads to inevitable differences between them. One critical intrinsic factor is the pulse transit time (PTT), which is the time required for the blood to travel from the heart to the peripheral site where the BF is measured. Therefore, it is expected that traveling through different blood vessel networks to different sites, such as the finger and ear, may affect the PTT and could affect the signals recorded and the information they provide. In addition, the two components of the ANS, the parasympathetic and sympathetic, have different roles in the heart and vasculature (Lombard and Cowley, 2012). While the SNS plays a dominant role in regulating vascular activity, the parasympathetic nervous system majorly contributes to cardiac activity. For example, many HRV indices, such as RMSSD, pNN50, and HF, reflect vagal tone. Other HRV indices, such as LF, reflect a mix of vagal and sympathetic activity (Laborde et al., 2017), suggesting a major role of vagal tone in HRV. However, peripheral BF and PRV may be affected by factors that affect HRV through the blood vessel network and by factors that directly affect vascular activity, such as the SNS (Task Force of the European Society of Cardiology and the North American Society of Pacing and Electrophysiology, 1996; Lombard and Cowley, 2012) or changes in the local vasculature (Heathers, 2013; Mejía-Mejía et al., 2020). Thus, variations in peripheral BF and PRV can come from different sources that do not affect HRV in the same way. This may also explain why some indices, such as ApEn, showed a trend of difference between PRV and HRV. This may be because PRV may be more complex than HRV, even during N3. Therefore, the differences between PRV and HRV require further investigation.

### 4.3 Determining different sleep stages using peripheral BF + PRV

Compared with ECG/HRV, peripheral BF/PRV can be measured in a much more convenient, straightforward, low-cost, and non-invasive way, such as using LDF or PPG, and at more recording sites. PPG can also be used to measure BF, but indirectly, which is not recommended according to some previous studies. On the other hand, LDF is the default method to measure BF because it relies on the Doppler shift directly related to the tissue BF, which could also explain the high sensitivity of LDF for measuring BF (Lindberg, Tamura, and Öberg, 1991; Wright, Kroner, and Draijer, 2006). PRV, derived from LDF or PPG, has been used to predict sleep stages with acceptable accuracy in previous studies (Dehkordi et al., 2014; Imtiaz, 2021). However, the peripheral BF signal itself is often ignored, and only some of the PRV features are used, which may be the reason for the lack of high accuracy. Some researchers have also tried to utilize both the peripheral BF or the corresponding PPG signal and PRV to automatically score sleep stages. However, the algorithms used in these studies extracted features that cannot be easily related to the physiological origins of the sleep stages (Uçar et al., 2018). The present study may have provided new features for determining different sleep stages and understanding their physiological origins. Although there are various models for sleep stage classification using PRV/HRV (Imtiaz, 2021), this study provides additional and essential sleep architecture information from peripheral BF for these models to use. With more related information, sleep staging will be expected to have higher accuracy.

### 4.4 The 0.2–0.3 Hz BF oscillations during sleep

HFn (0.15–0.40 Hz) reflects respiratory sinus arrhythmia used to measure parasympathetic (vagal) activity, and the 0.2–0.3 Hz oscillations are within the HFn frequency band. This frequency band is consistent with the so-called Traube-Hering waves, which refer to blood pressure oscillations in time with breathing (Barnett et al., 2020). A plausible explanation is that the band may reflect the respiratory modulation of peripheral BF and PRV/HRV (see Supplementary Figure S4). Both finger- and ear-BF showed an apparent peak power in the 0.2–0.3 Hz band, which increased with the deepening of sleep, e.g., from N1 to N3. In addition, the relative power in the 0.2–0.3 Hz band of both finger- and ear-BF correlated with that of respiratory activity. Previous studies have also reported that respiratory events modulate the pulse wave amplitude of peripheral BF more than ECG/HRV (Haba-Rubio et al., 2005; Khandoker, Karmakar, and Palaniswami, 2011). PRV can reflect the coupling effect between respiration and vasculature. However, although there have been studies on the effects of respiration around 0.2–0.3 Hz on cardiovascular activity (Krasnikov et al., 2013; Tankanag, Krasnikov, and Mizeva, 2020), no studies have been conducted to investigate this band of oscillations during sleep, especially by comparing it among sleep stages. The results of the present study showed that oscillations occur in the 0.2–03 Hz band during sleep and that sleep stages modulate these oscillations. Specifically, the 0.2–0.3 Hz oscillations were largest during N3 (deep sleep) and were reflected in peripheral BF, PRV, and HRV.

Sleep affects breathing patterns and ANS activity, thus affecting peripheral BF and PRV. For example, during N3, both peripheral BF and PRV showed 0.2–0.3 Hz oscillations, and the 0.2–0.3 Hz oscillations of peripheral BF were correlated with those of AF. Conversely, changes in breathing patterns and ANS activity may modulate sleep. Supposing the 0.2–0.3 Hz oscillations can be affected by external stimulation, such as sensory stimulation (e.g., rocking), this pathway may modulate sleep. A recent study conducted using 0.25 Hz rocking stimulation successfully entrained spontaneous neural oscillations with benefits for sleep and memory (Perrault et al., 2019). Several other similar studies also focused on rocking frequency within 0.2–0.3 Hz (Shibagaki et al., 2017; Omlin et al., 2018; van Sluijs et al., 2020), which is why we proposed focusing on this narrow band instead of on HF. The present study provides consistent evidence for using stimulation of approximately 0.25 Hz to facilitate sleep. However, the causal effects of stimulation in the 0.2–0.3 Hz band on peripheral BF activity during sleep have not been investigated yet; thus, future studies are needed to clarify this aspect.

### 4.5 Limitations of this study

Although measurement of peripheral BF/PRV is much more convenient, factors that affect PRV rather than HRV include interruptions during recording, noise/artifacts in the recorded signals, errors in detecting the fiducial points, and physiological factors such as the PTT and changes (or respiration factors leading to changes) in blood pressure. The recording interruptions and noise/artifacts mainly come from unstable attachment of the sensors to body areas (e.g., ear concha) and are related to body movement. Several measurements and a certain number of epochs from individual data were excluded from analysis because of noise/artifacts during acquisition (Supplementary Table S1) and interruptions during recording. Body movements and posture can easily affect the right-index finger and right-ear concha sites. Thus, the first limitation of this study was the movement artifacts that affect the quality of peripheral BF and PRV data. The noise/artifacts problem may be solved with further development of these sensors, especially the ear sensor. Future device designs should increase the signal-to-noise ratio and robustness to recording interruptions. Further, owing to the noise/artifacts, the quality of fiducial point detection was affected. Nevertheless, despite the immerits of data integrity and quality, this study still provided the possibility for PRV to be equivalent to HRV in some parameters and the possibility that peripheral BF provides additional sleep architecture information.

The second limitation of this study was the evaluation of systolic peaks to detect the fiducial point for PRV as an analogy to the actions of R peaks for HRV. However, this method shows the lowest agreement between PRV and HRV. This may not have greatly affected this study, because finger-PRV showed considerable consistency with HRV. However, ear-PRV must have been significantly affected. Even during preprocessing, we discovered that detecting the fiducial point was complex for many epochs of ear-BF because of noise/artifacts. Therefore, external factors such as technical design and noise/artifacts, including movement artifacts that affected peripheral BF + PRV, and not intrinsic factors, such as the nature of peripheral BF and ECG, may have affected the consistency between the two.

The third limitation is that we only recruited healthy participants in this study. No patients, for example, those with sleep apnea, were included. Thus, this study cannot be directly compared with previous studies on patients (Khandoker et al., 2011; Liu et al., 2017). However, the healthy sleep status of the participants was self-reported, which does not exclude the possibility that there may have been undiagnosed sleep disorders present. Further, although participants were told to refrain from taking alcohol and medicines on the day of the experiment, it is possible that they may be under medication or taking supplements that influence sleep, ANS function, and peripheral function. Thus, future studies should include more strict screening, especially when comparing healthy populations with patients.

The fourth limitation is that we only conducted the experiment during the nighttime. HRV is modulated by the circadian rhythm (Stein, 2007), which suggests that the dynamics of peripheral BF and PRV during sleep stages observed in this study may differ between daytime naps and nighttime sleep. Nevertheless, the present study has proved that peripheral BF + PRV provides much information about sleep stages, which may be more than that provided by HRV alone, supporting the prospect of peripheral BF + PRV as a new biomarker (Yuda et al., 2020), although this conclusion should be further explored in future studies.

The fifth limitation is the statistical power to detect differences in HRV, PRV, and peripheral BF indices between stages. Several factors affected this, including individual differences in the sample, sample size, noise/artifacts, and data integrity as discussed above. For this reason, Bayesian analysis was applied, parallel to frequentist tests for obtaining more information, especially in the case of non-significant differences, which is a merit of Bayesian analysis.

## 5 Conclusion

In conclusion, the peripheral BF signal and derived IBI signal (for the calculation of PRV) provide considerable information about the sleep state, specifically, the sleep stages. Time-domain and frequency-domain parameters of peripheral BF, and time-domain, frequency-domain, and non-linear PRV indices provide ample information about the sleep stages, possibly more than HRV alone. Accordingly, peripheral BF + PRV should be considered a new biomarker instead of a surrogate of ECG/HRV, and its potential for monitoring and predicting sleep state should be further explored.

## Data Availability

The original contributions presented in the study are included in the article/Supplementary Materials, further inquiries can be directed to the corresponding author.
