# Supplementary material for: Peripheral blood flow estimated by laser doppler flowmetry provides additional information about sleep state beyond that provided by pulse rate variability
Source: Front Physiol. 2023 Jan 26;14:1040425. doi: 10.3389/fphys.2023.1040425 (PMC9908953; doi:10.3389/fphys.2023.1040425)
Supplement: Supplementary file 1 [file DataSheet1.docx]

Peripheral blood flow estimated by laser doppler flowmetry provides additional information about sleep state beyond that provided by pulse rate variability

**Table S1. Usability of data (epochs) for ECG, finger-BF, and ear-BF across the sleep stages**

|  |  |  | |  |  |  |  |  |
| --- | --- | --- | --- | --- | --- | --- | --- | --- |
| Data | Non-NaN epochs  / All 320 epochs (%) | Available epochs / Non-NaN epochs (%) | | | | | | |
|  |  | All | | Wk | N1 | N2 | N3 | REM |
| ECG | 68.72 ± 12.07 | 92.34 ± 5.97 | 74.30 ± 19.15 | | 92.06 ± 17.20 | 95.66 ± 6.21 | 93.55 ± 11.66 | 94.15 ± 8.83 |
| Finger-BF | 68.18 ± 12.91 | 72.37 ± 22.58 | | 58.84 ± 28.17 | 74.30 ± 31.33 | 72.79 ± 24.13 | 80.67 ± 23.54 | 66.28 ± 27.87 |
| Ear-BF | 67.75 ± 13.78 | 56.79 ± 34.36 | | 52.14 ± 32.87 | 55.48 ± 39.28 | 56.71 ± 35.94 | 61.20 ± 39.35 | 52.70 ± 38.04 |
|  |  |  | |  |  |  |  |  |

Note: The percentage here is the average of all available individual percentages.

NaN, not a number; Wk, wakefulness; N1–3, non-rapid-eye-movement sleep 1–3; REM, rapid-eye-movement sleep; ECG, electrocardiogram; BF, blood flow.

**Table S2. Wrongly detected epochs for ECG, finger-BF, and ear-BF across the sleep stages**

| Data | Participants with wrong detections   / Available participants | Wrongly detected epochs / Available epochs after preprocessing (%) | | | | | |
| --- | --- | --- | --- | --- | --- | --- | --- |
|  |  | All | Wk | N1 | N2 | N3 | REM |
| ECG | 1/45 | 0.02 ± 0.12 | 0.07 ± 0.44 | 0.00 ± 0.00 | 0.00 ± 0.00 | 0.00 ± 0.00 | 0.00 ± 0.00 |
| Finger-BF | 4/38 | 0.50 ± 1.71 | 0.00 ± 0.00 | 0.38 ± 2.18 | 0.59 ± 2.46 | 0.63 ± 3.44 | 0.32 ± 1.96 |
| Ear-BF | 39/42 | 6.81 ± 14.15 | 9.15 ± 20.62 | 7.04 ± 20.40 | 6.02 ± 16.57 | 6.00 ± 18.23 | 7.47 ± 19.48 |

Note: The percentage here is the average of all available individual percentages.

Wk, wakefulness; N1–3, non-rapid-eye-movement sleep 1–3; REM, rapid-eye-movement sleep; ECG, electrocardiogram; BF, blood flow.

**Table S3. PRV/HRV indices selected for analysis**

| MeanIBI | Mean of inter-beat intervals corresponding to R-to-R intervals or pulse-to-pulse of blood flow intervals |  |
| --- | --- | --- |
| Time-domain indices | | |
| SDNN | Standard deviation of all the normal IBIs (normal-to-normal [NN] intervals) |  |
| RMSSD | Root mean square of successive differences between adjacent NN intervals |  |
| pNN50 | Percentage of pairs of adjacent NN intervals differing by more than 50 ms |  |
| Frequency domain indices | | |
| LFn | Normalized low frequency (0.04–0.15 Hz) power: LF/(LF+HF) |  |
| HFn | Normalized high frequency (0.15–0.40 Hz) power: HF/(LF+HF) |  |
| LF/HF | Ratio of LF to HF |  |
| Non-linear measurements | | |
| ApEn | Approximate entropy |  |
| DFA1 | Detrended fluctuation analysis to measure short-range fluctuations (4 to 12 beats) |  |
| DFA2 | Detrended fluctuation analysis to measure long-range fluctuations (13 to 64 beats) |  |

Note: PRV, pulse rate variability; HRV, heart rate variability; R, R peaks; IBIs, inter-beat intervals; LF, low frequency power; HF, high frequency power.

**Table S4. Evidence categories for the Bayes factor** $\boldsymbol{B}_{\boldsymbol{10}}$

| Bayes factor $B_{10}$ | Interpretation |
| --- | --- |
| > 100 | Extreme evidence for H1 |
| 30–100 | Very strong evidence for H1 |
| 10–30 | Strong evidence for H1 |
| 3–10 | Moderate evidence for H1 |
| 1–3 | Anecdotal evidence for H1 |
| 1 | Insufficient evidence for either H1 or H0 |
| 0.33–1 | Anecdotal evidence for H0 |
| 0.1–0.33 | Moderate evidence for H0 |
| 0.03–0.1 | Strong evidence for H0 |
| 0.01–0.03 | Very strong evidence for H0 |

Note: H1, the alternative hypothesis; H0, the null hypothesis; $B_{10}$, Bayes factor for H1 to H0.

**Frequentist test for BF, AF, HRV, and PRV parameters across the sleep stages**

We investigated the changes in each BF, AF, HRV, and PRV parameter across the sleep stages separately for each recording site (heart, finger, or ear) using the frequentist tests. For indices that met the normality criteria, we performed parametric analysis using one-way (stages: Wk, N1, N2, N3, and REM) frequentist repeated measures analysis of variance (F-RMANOVA) with Greenhouse–Geisser correction for the violation of sphericity. Post-hoc test was conducted when the main effect of the sleep stage was significant. We conducted a non-parametric analysis using Friedman's test with the Conover's post-hoc test for pairwise comparisons for indices that did not meet the normality criteria. The Bonferroni method was used for multiple (ten in total) comparisons corrections. Statistical significance was set at *p* < 0.05.


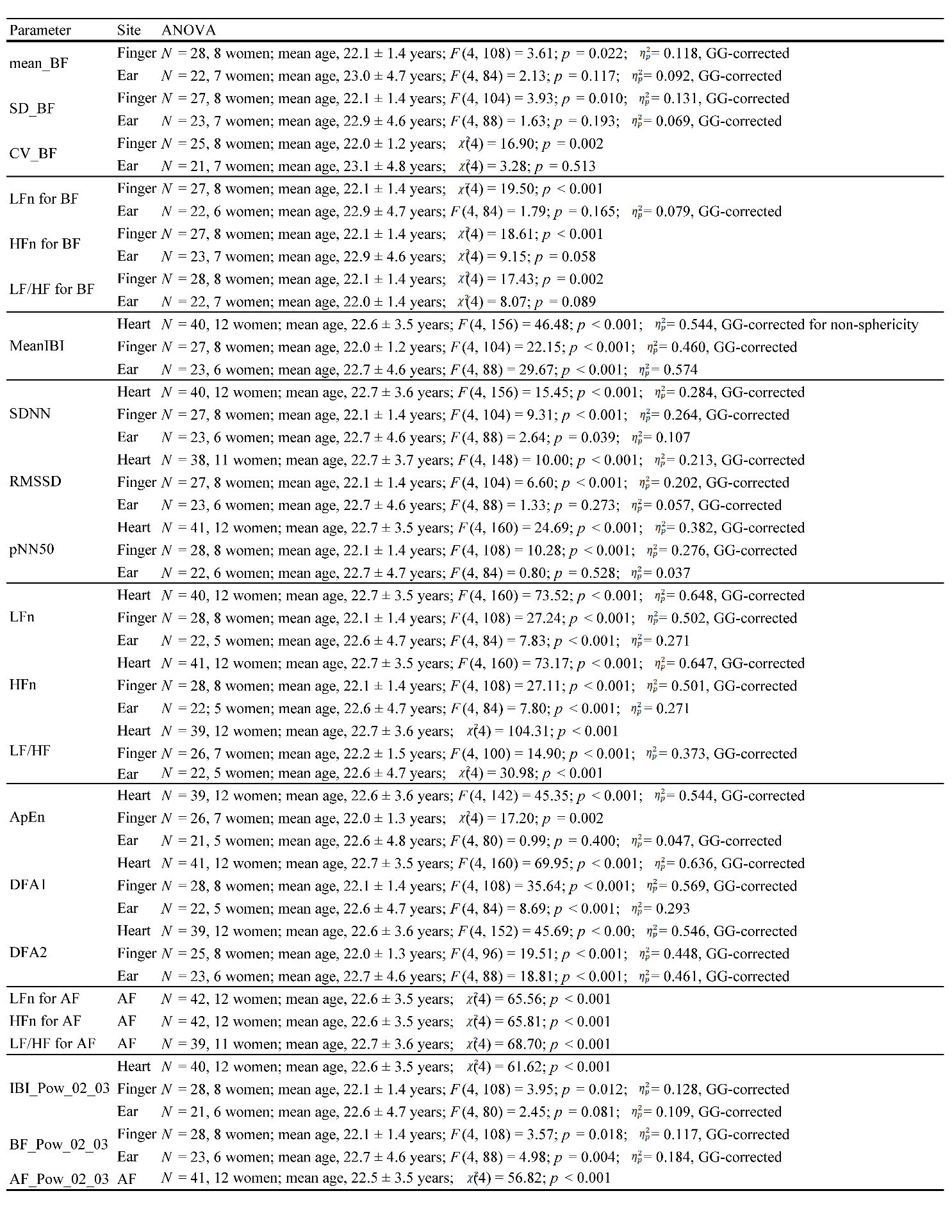
**Table S5. The results of frequentist tests for the effect of sleep stages on the BF/AF/HRV/PRV indices measured at each recording site**

Note: GG-corrected: Greenhouse–Geisser correction for the violation of sphericity.

HRV, heart rate variability; PRV, pulse rate variability; BF, blood flow; Wk, wakefulness; N1–3, non-rapid-eye-movement sleep 1–3; REM, rapid-eye-movement sleep; MeanIBI, mean of inter-beat intervals corresponding to R-to-R intervals or pulse-to-pulse of blood flow intervals; SDNN, standard deviation of all the normal-to-normal intervals; RMSSD, root mean square of successive differences between the adjacent normal-to-normal intervals; pNN50, percentage of pairs of the adjacent normal-to-normal intervals differing by more than 50 ms; LFn, normalized low-frequency power; HFn, normalized high frequency power; ApEn, approximate entropy; DFA, detrended fluctuation analysis; SD, standard deviation; CV, coefficient of variance; BF_Pow_02­_03, power of BF data in 0.2–0.3 Hz; IBI_Pow_02­_03, power of inter-beat intervals data in 0.2–0.3 Hz; AF, airflow; AF_Pow_02­_03, power of airflow data in 0.2–0.3 Hz.


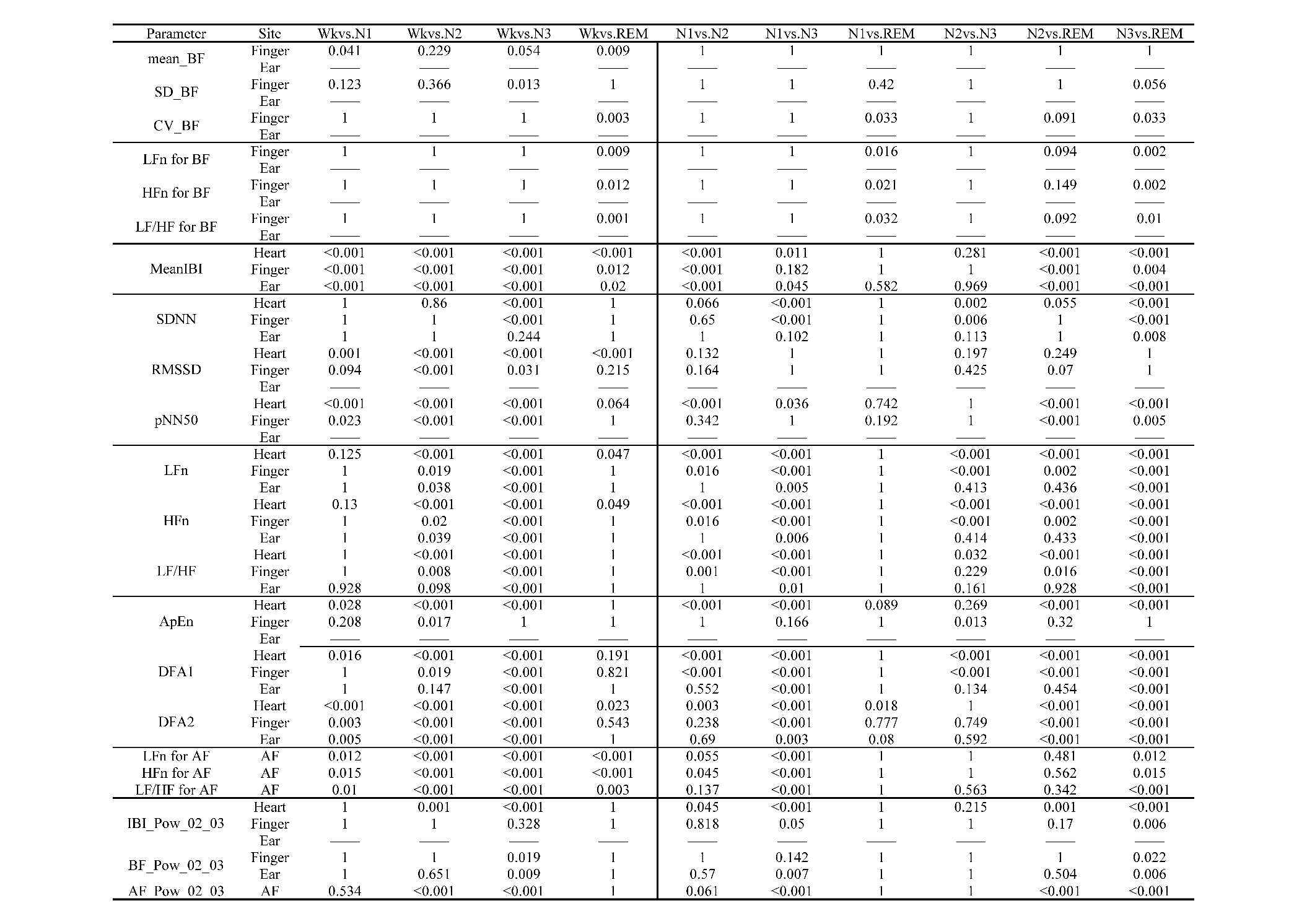
**Table S6. The results (p values) of post-hoc tests of different pairs of sleep stages for the BF/AF/HRV/PRV indices measured at each recording site**

Note: HRV, heart rate variability; PRV, pulse rate variability; BF, blood flow; Wk, wakefulness; N1–3, non-rapid-eye-movement sleep 1–3; REM, rapid-eye-movement sleep; MeanIBI, mean of inter-beat intervals corresponding to R-to-R intervals or pulse-to-pulse of blood flow intervals; SDNN, standard deviation of all the normal-to-normal intervals; RMSSD, root mean square of successive differences between the adjacent normal-to-normal intervals; pNN50, percentage of pairs of the adjacent normal-to-normal intervals differing by more than 50 ms; LFn, normalized low-frequency power; HFn, normalized high frequency power; ApEn, approximate entropy; DFA, detrended fluctuation analysis; SD, standard deviation; CV, coefficient of variance; BF_Pow_02­_03, power of BF data in 0.2–0.3 Hz; IBI_Pow_02­_03, power of inter-beat intervals data in 0.2–0.3 Hz; AF, airflow; AF_Pow_02­_03, power of airflow data in 0.2–0.3 Hz.





**Figure S1**. Data preprocessing flowchart.

* Correction is only for BF IBIs, not ECG IBIs. The ECG IBIs were found to be of good quality during manual removal of wrong peak detections.


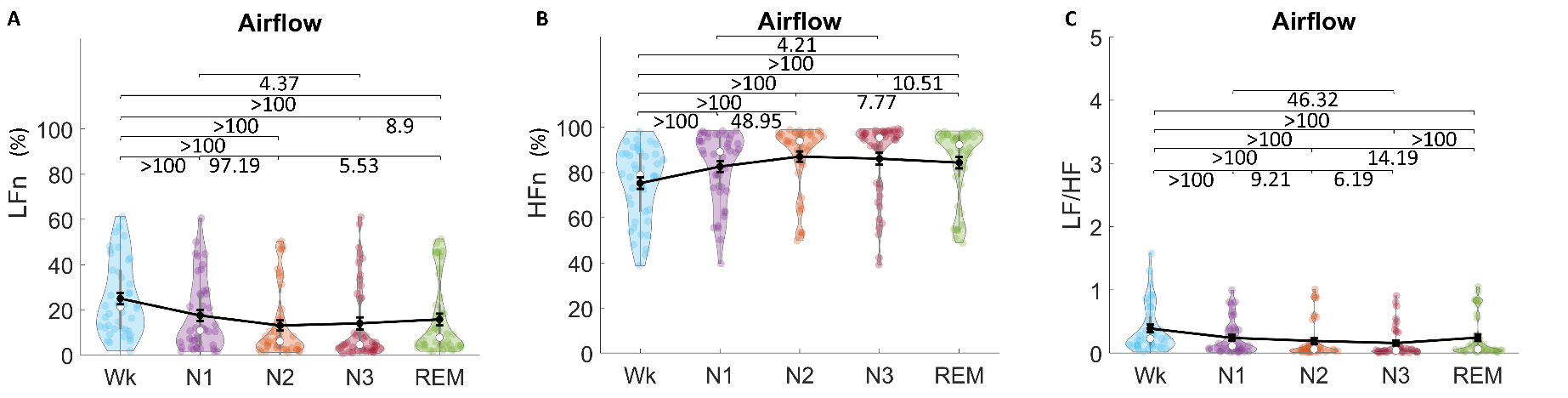


**Figure S2**. LFn (A), HFn (B), and LF/HF (C) of AF across the different sleep stages. The violin plot with dots shows the distribution of the individual data points. The line chart with error bars shows the group mean and the ± 1 standard error of the mean.

The numerical values are the Bayes factors, which show different levels of evidence (see Table S4) against the H0 of no difference between pairs of sleep stages. Bayes factors < 1 are not listed.

LFn, normalized low-frequency power; HFn, normalized high-frequency power; LF, low-frequency power; HF, high-frequency power; BF, blood flow; AF, airflow.


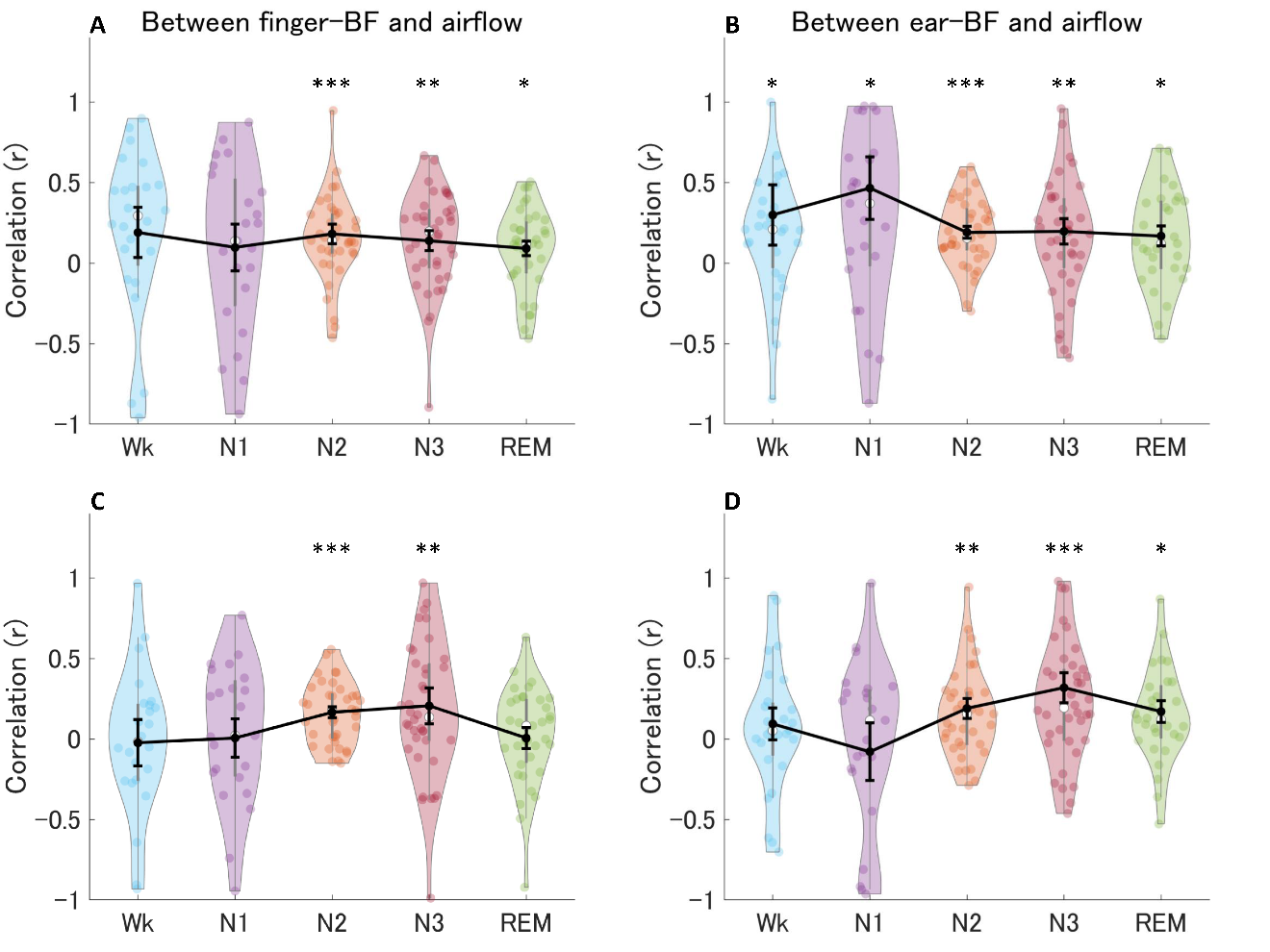


**Figure S3.** The correlations between the relative band power of 0.2–0.3 Hz for finger-BF and AF (A), and ear-BF and AF (B), as well as the correlations between the peak frequency within 0.2–0.3 Hz for finger-BF and the peak frequency within 0.15–0.4 Hz for AF (C), and ear-BF and AF (D). The violin plot with dots shows the distribution of the individual data points. The black line chart with error bars shows the inverse Fisher z-transformation of the group mean of Z coefficients (transformed from r) and the inverse Fisher z-transformation of the ± 1 standard error of the mean.

**p* < 0.05; ***p* < 0.01; ****p* < 0.001; BF, blood flow; AF, airflow.

**Methods used to obtain the results in Figures 5C and F and in Figure S3**

Figure S3 shows the correlations between the relative band power of 0.2–0.3 Hz for finger-/ear-blood flow (BF) and airflow (AF), as well as the correlations between the peak frequency within 0.2–0.3 Hz for BF and the peak frequency within 0.150.4 Hz for AF, across epochs in each sleep stage.

Similar to the analysis of BF, 90-s epochs of AF from each individual were preprocessed using the customized program in MATLAB (The MathWorks Inc., Natick, MA, USA). The epochs with the largest amplitude beyond the three standard deviations of the median value of the highest amplitudes were excluded to remove epochs with significant artifacts. In addition, epochs with peaks of the waveform of AF (filtered using the default band-pass filter of 0–0.5 Hz embedded in the FieldTrip toolbox [22] beyond the three standard deviations of the median value of all the peaks were also excluded to ensure the quality of the beat signals was optimal.

The power spectra of AF were also analyzed using the “plomb” function in MATLAB. The band power of 0.2–0.3 Hz was extracted, normalized by dividing it by the high-frequency power (as well as the normalized high- and low-frequency power, and low-/high- frequency power of AF, see Figure S2), and compared. The individual AF values larger than three standard deviations of the group means were set as missing values. Participants with missing values in any five stages were excluded from the group analysis.

The correlations between the relative band power of 0.2–0.3 Hz for BF and AF, and between the peak frequency within 0.2–0.3 Hz for BF and that within 0.15–0.4 Hz for AF, were calculated across epochs in each sleep stage for each individual. The results were then subjected to group analysis independently for each sleep stage.

For comparisons of the relative band power of 0.2–0.3 Hz for AF between different sleep stages, the statistical analysis was similar to BF (see Figures 5C and F for the results). For the significance test of the correlation coefficients, they were first subjected to Fisher z-transformation using the “atanh” function in MATLAB. Then, a one-sample t-test was conducted for the group coefficients that met the normality, whereas the Wilcoxon signed-rank test was conducted for those that did not meet the normality. The tests were performed independently for each sleep stage (see Figure S3 for the results).


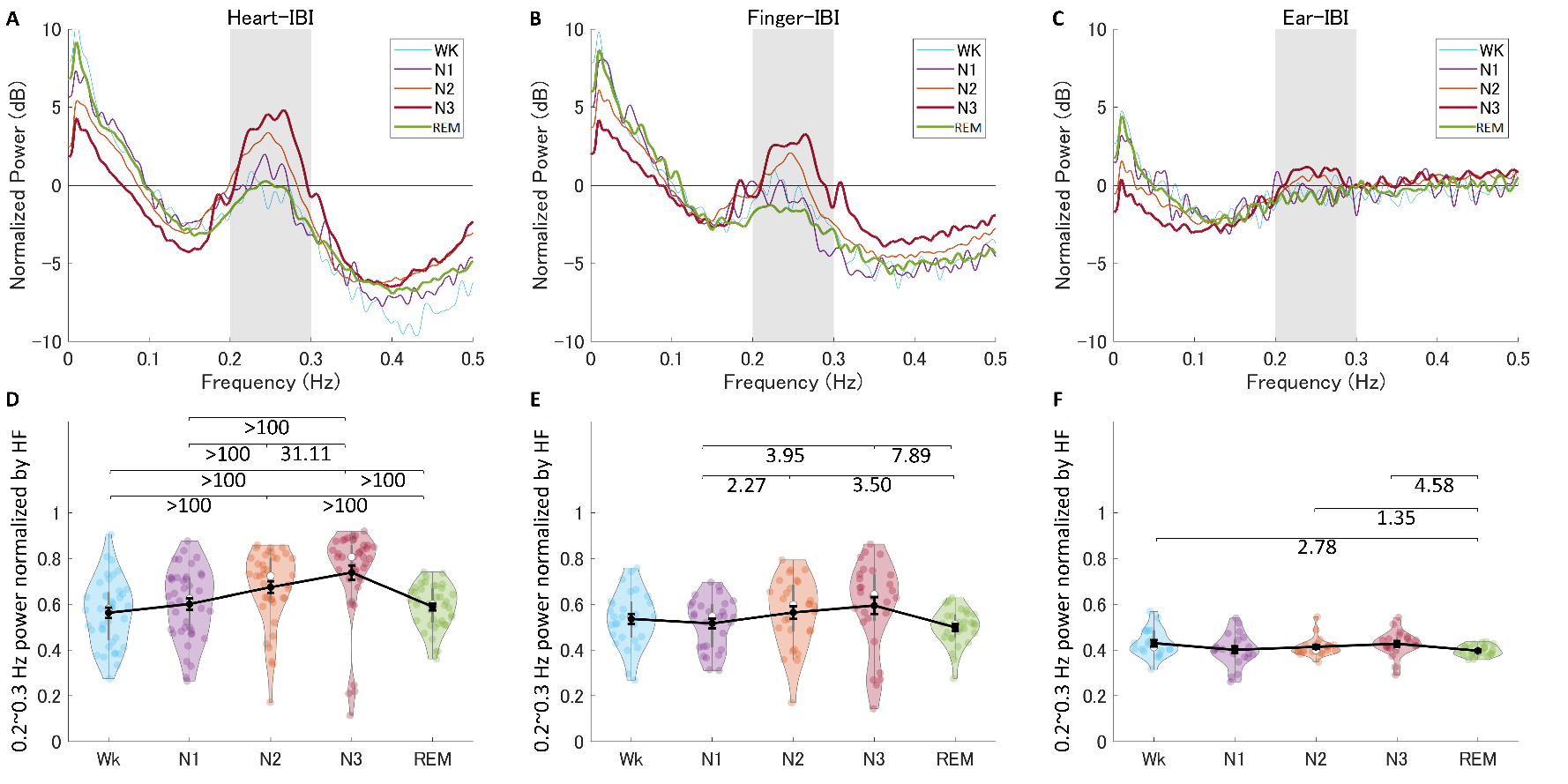


**Figure S4**. The normalized power spectra and the normalized power of the 0.2–0.3 Hz band of HRV, finger-PRV, and ear-PRV across the different sleep stages. (A-C) The normalized power spectra for HRV (A), finger-PRV (B), and ear-PRV (C). (D-F) The normalized power of the 0.2–0.3 Hz band for HRV (D), finger-PRV (E), and ear-PRV (F). There was a linear trend of power increase in the 0.2–0.3 Hz band with the deepening of sleep from N1 to N3 for finger-PRV, ear-PRV, and HRV. The violin plot with dots shows the distribution of the individual data points. The line chart with error bars shows the group mean and the ± 1 standard error of the mean.

The numerical values represent the Bayes factors, which show different levels of evidence (see Table S4) against the H0 of no difference between pairs of sleep stages. Bayes factors < 1 are not listed.

HRV, heart rate variability; PRV, pulse rate variability; IBI, inter-beat interval; HF, high-frequency power.

As mentioned in the Methods section, we also investigated the (normalized) power spectra of the IBI signals derived from the BF and ECG signals (IBI_Pow_02_03). For the IBI_Pow_02_03 of HRV/PRV, which was normalized by dividing it by the HF for each recording site, the results (Figure S4) showed the patterns for HRV, finger-PRV, and ear-PRV across sleep stages were all similar. For HRV, finger-PRV, and ear-PRV, normalized IBI_Pow_02_03 was higher in NREM (N2 and N3) sleep than in REM sleep; however, the evidence for ear-PRV was weak. For NREM sleep, IBI_Pow_02_03 was higher in N3 than in N1, and in N2 than in N1, for both HRV and finger-PRV; however, the evidence for finger-PRV was weaker. Trend analysis revealed a linear trend for finger-IBI (p < 0.001) and ear-BF (p = 0.010); however, ear-IBI provided weaker evidence.
